# Spatial Variation in Soil Properties among North American Ecosystems and Guidelines for Sampling Designs

**DOI:** 10.1371/journal.pone.0083216

**Published:** 2014-01-17

**Authors:** Henry Loescher, Edward Ayres, Paul Duffy, Hongyan Luo, Max Brunke

**Affiliations:** 1 Science Team, National Ecological Observatory Network (NEON), Boulder, Colorado, United States of America; 2 Institute of Arctic and Alpine Research (INSTAAR), University of Colorado, Boulder, Colorado, United States of America; 3 Natural Resource Ecology Laboratory, Colorado State University, Fort Collins, Colorado, United States of America; 4 Neptune and Company Inc., Lakewood, Colorado, United States of America; DOE Pacific Northwest National Laboratory, United States of America

## Abstract

Soils are highly variable at many spatial scales, which makes designing studies to accurately estimate the mean value of soil properties across space challenging. The spatial correlation structure is critical to develop robust sampling strategies (*e.g.*, sample size and sample spacing). Current guidelines for designing studies recommend conducting preliminary investigation(s) to characterize this structure, but are rarely followed and sampling designs are often defined by logistics rather than quantitative considerations. The spatial variability of soils was assessed across ∼1 ha at 60 sites. Sites were chosen to represent key US ecosystems as part of a scaling strategy deployed by the National Ecological Observatory Network. We measured soil temperature (T_s_) and water content (SWC) because these properties mediate biological/biogeochemical processes below- and above-ground, and quantified spatial variability using semivariograms to estimate spatial correlation. We developed quantitative guidelines to inform sample size and sample spacing for future soil studies, *e.g.*, 20 samples were sufficient to measure T_s_ to within 10% of the mean with 90% confidence at every temperate and sub-tropical site during the growing season, whereas an order of magnitude more samples were needed to meet this accuracy at some high-latitude sites. SWC was significantly more variable than T_s_ at most sites, resulting in at least 10× more SWC samples needed to meet the same accuracy requirement. Previous studies investigated the relationship between the mean and variability (*i.e.*, sill) of SWC across space at individual sites across time and have often (but not always) observed the variance or standard deviation peaking at intermediate values of SWC and decreasing at low and high SWC. Finally, we quantified how far apart samples must be spaced to be statistically independent. Semivariance structures from 10 of the 12-dominant soil orders across the US were estimated, advancing our continental-scale understanding of soil behavior.

## Introduction

Researchers interested in measuring the mean value for a soil property across space must make decisions about the number of samples required and how far apart they should be spaced. These decisions are inherently dependent on the variance structure and spatial independence of the data, but quantitative guidelines to inform these decisions are unavailable at most sites. For instance, intuitively researchers know that a large number of widely-spaced samples would be more likely to better measure the spatial mean of a soil property than a few samples all located close to one another. But it is typically not clear what “a large number” or “widely-spaced” means. Moreover, since increasing the sample size consumes resources (*e.g.*, time, money, and analytical capacity), it is important that sufficient samples are collected to meet the accuracy requirement to answer a specific question without wasteful over-sampling. Likewise, choosing an appropriate distance between samples is important for two reasons; 1) samples should ideally be spaced sufficiently far apart to avoid correlation and pseudoreplication in measurements at a given scale [1], thereby maximizing the amount of information provided by a limited number of samples, and 2) spacing between samples should be minimized to constrain costs, which typically increase with distance.

Here, we focus on sampling strategies designed to estimate the spatial mean value of a soil property with the smallest possible sample size and sample spacing. However, it is also important to note that other goals also exist that require different sampling designs, *e.g.*, redundant sampling (*i.e.*, field duplicates) involving two or more measurements at the same location to aid error detection (*e.g.*, sensor malfunction) and increase confidence in the data, while varying the distance between adjacent samples can be useful when characterizing the spatial variance structures.

The number of samples and sample spacing is rarely justified in research papers or presentations. In many, if not most studies, decisions relating to the sampling strategy appear to be based on a combination of subjective opinion, repeating previously used sampling designs (often from a different ecosystem or in relation to a different soil property), and available resources. There are only a few methodological articles that discuss sampling designs recognize the importance of choosing an appropriate sample size and sample spacing, and provide only vague- or no recommendations at all [2]–[4]. The advice often given is to conduct a preliminary spatial variability study (of soil properties) at the research site to determine the number of samples and sample size required to achieve a given accuracy [4], [5]. While this is good advice, it is rarely followed, presumably due to a lack of time, money, and/or an appreciation of its importance.

Soils are highly variable and embody systematic (*e.g.*, soil forming processes) and random sources of variability across space [6], [7]. Some studies have used the coefficient of variation to assess this variability at a given site, and used in sample size analyses [8]–[14]. Geostatistical techniques, in particular semivariograms, have increasingly been used over the last few decades to characterize spatial variation in soil properties [15], [16]. Semivariograms provide two useful components for designing a robust sampling strategy: 1) an estimate of the variance (*i.e.*, the semivariogram sill), which can be used to inform sample size in future studies; and 2) an estimate of the minimum distance required for samples to be considered spatially independent (*i.e.*, the semivariogram range), which can be used to inform sample spacing (Figure S1). We emphasize that the use of sample size analyses in this paper is to inform decisions relating to future studies, not to assess the power of a sampling design retrospectively, which may be fundamentally flawed [17].

Site-specific studies that have assessed spatial variation in soil properties have shown that spatially structured variability is ubiquitous. For instance, Robertson et al. [11] studied variation in soil physical, chemical, and biological properties across an agricultural field in Michigan, USA. Almost every soil property exhibited spatially structured variation, which in most cases accounted for the majority of the variability that was observed Robertson et al. [11], and indicated that measurements of soil properties at one location could be used to predict values for those properties up to ∼60 m away (*i.e.*, the range). Similar findings have been reported elsewhere, across a range of ecosystems and soils [9], [12], [18]–[24], although there are a few exceptions, with some properties exhibiting little or no spatial dependence at the scale studied [9], [11], [19], [20]. The identification of spatial patterns in soil properties among sites, in particular at larger continental scales has been hampered, because most studies have investigated these quantities at a single site.

The few cross-site comparisons studies have focused on soil moisture variability located only within a small region with relatively narrow ecoclimatic variability [13], [14], [25] or, if they spanned larger scales, involved only a few sites [26] or combined data from different studies with widely differing designs *e.g.*, spatial extent, physical quantities, sampling depth, seasonal timing, measurement technique, etc. [12], [27]–[30]. Moreover, previously published data are often unavailable for additional analyses. A single study that employs a uniform sampling and analysis approach, *i.e.*, the above mentioned quantities, as well as being representative of site characteristics (*e.g.*, ecosystem structure, soil type, etc), made under similar environmental conditions for a wide range of ecosystems is needed for meaningful cross-site comparisons and to develop quantitative sampling guidelines that can inform sampling strategies in future soil studies.

Here, we studied the spatial variation in soil properties using a consistent approach at 60 sites throughout the USA and developed quantitative relationships to provide: 1) an ecological understanding of spatial variability in soils, and 2) guidance on sampling designs for future soil studies. We studied local scale (∼1 ha) variation in soil temperature (T_s_) and soil water content (SWC) at sites that included representatives from all major terrestrial ecosystem types, soil types, and climates found in the USA. Soil temperature and moisture were used as proxies for other soil properties because they can be measured quickly and accurately in the field, they are major drivers of soil biotic and biogeochemical properties and processes, their spatial pattern integrates the spatial pattern of ecosystem canopy structure and has been shown to correlate with spatial patterning of other soil properties at the local scale, and they are increasingly recognized as important variables in understanding land-atmosphere interactions *e.g.*, soil moisture is recognized as a Global Climate Observing System Essential Climate Variable [9], [11], [31]–[36]. The sampling scale (∼1 ha) corresponds to many ecosystem-level soil studies and is the basis of many large emergent observatories (National Ecological Observatory Network, NEON, www.neoninc.org; Integrated Carbon Observing System, www.icos-infrastructure.eu). We used these data to further examine the relationship between the spatial structure of variability in soil properties and site characteristics at broader landscape- to continental scales (*e.g.*, latitude, climate, ecosystem type, soil type, and vegetation structure). Lastly, we used analyses to estimate the sample size required to accurately estimate the spatial mean T_s_ and SWC at each site.

This study was made to inform the sensor-based soil plot designs at NEON sites. In addition, several hypotheses were tested. We hypothesized that;

1. the spatial variability in soil properties would be associated with variability in ecosystem structure, *e.g.*, we expect open canopy ecosystems, which exhibit large spatial variability in vegetation height (*e.g.*, savannas) to have larger variability in soil properties than closed canopy ecosystems,
2. agricultural ecosystems would exhibit lower spatial variability in soil properties than other ecosystems, since management activities (*e.g.*, tillage and irrigation) aim to homogenize properties,
3. younger soils (*e.g.*, inceptisols) would exhibit both lower spatial variability and have smaller range values than older soils (*e.g.*, ultisols). We expected that some soil properties (*e.g.*, soil texture) that influence the spatial structure of variability are controlled by processes that operate over long temporal scales (*e.g.*, weathering),
4. the spatially structured variability (*e.g.*, semivariogram range, nugget, and sill), as well as the sample size required to accurately estimate the mean, would be positively correlated for soil temperature (T_s_) and soil water content (SWC), becuase some underlying site characteristics (*e.g.*, canopy gaps, etc.) are likely to influence both variables,
5. some forms of semivariograms may be more common than other forms, *e.g.*, , that small semivariogram range values would be more commonly associated with small semivariogram sills than large sills, and
6. variability would be larger for SWC than T_s_ since water is more mobile than heat and sensitive to microtopography, as well as being actively transported by plants.

We also aimed to develop empirically-based rules that could be used by other researchers to guide sampling designs to accurately estimate the spatial mean of soil properties for future studies.

## Methods

### Site descriptions and data collection

The study was conducted at 60 NEON sites (Table 1). These sites were chosen for landscape level representativeness using the approaches of Hargrove and Hoffman [37], [38], which divided the USA into 20 eco-climatic domains. Within each domain, 3 representative site were chosen; 1 wildland (*i.e.*, relatively undisturbed) site and typically 2 sites that address key ecological issues for that domain (*e.g.*, urbanization, land management, advance of invasive species, or climate change) [39], [40]. Measurements at these sites included ecosystem scale (*i.e.*, 10∧3 to 10∧6 m^2^) tower-based estimates of eddy covariance [41]–[44], and the long term sensor-based soil measurements are required to be made in the flux footprint of the tower, *i.e.*, at the flux scale [45]–[47] and occur on locally (co-) dominant soil series. No sampling occurred requiring any sampling permits (re soil sampling), no human sampling occurred, nor the use or impact to any endangered or protected animals were involved in this study. The sampling area for this study corresponded to the expected location of NEON's long term sensor-based soil measurements at each site. We received permission to access and to measure soil properties by the owner at all sites, and no soil samples were taken (hence not requiring a sampling permit of any kind). Two National Park Service sites required a research permit to gain access to the site (see Table 1).

**Table 1:** Site characteristics, where Lat = Latitude, Long = Longitude, El = Elevation, Temp = Mean Annual Air Temperature, Precip = Mean annual Bulk Precipitation, CH = Canopy Height, CS = Canopy Structure, CL = closed, SS = short stature, O = open, SO = semi-open.

Site	Lat (°)	Long (°)	El (m.a.s.l)	Temp (°C)	Precip (mm)	CH (m)	Ecosystem type	CS	Soil order	Owner
Harvard Forest	42.54	−72.17	348	7	1066	26.0	Temperate deciduous forest	CL	Spodosol	Harvard U
Burlington	42.52	−71.18	38	9	1095	24.0	Temperate deciduous forest	CL	Inceptisol	Town of Burlington MA
Bartlett Exp. Forest	44.06	−71.29	273	7	1219	23.0	Temperate deciduous forest	CL	Spodosol	USDA FS
Blandy Farm	39.06	−78.07	182	12	972	1.0	Shrubland	SS	Ultisol-Alfisol	U Virginia
Smithsonian CRC (SCBI)	38.89	−78.14	355	13	1057	35.0	Temperate deciduous forest	CL	Alfisol	Smithsonian Institution
Smithsonian SERC	38.89	−76.56	10	13	1097	38.0	Temperate deciduous forest	CL	Ultisol	Smithsonian Institution
Ordway-Swisher BS	29.69	−81.99	48	20	750	23.0	Temperate coniferous forest	O	Entisol	U Florida Foundation
J. Jones Ecological Research station	31.19	−84.47	47	19	1344	27.0	Temperate coniferous forest	CL	Ultisol	Robert W. Woodruff Foundation
Disney Wilderness Preserve	28.13	−81.44	20	22	1219	1.5	Grassland	SS	Spodosol	Nature Conservancy
Guanica State Forest	17.97	−66.87	126	23	840	10.0	Tropical deciduous forest	CL	Aridisol	State Forest, Dept. Natural and Environmental Resources, Puerto Rico
Lajas Ag. Exp. St.	18.02	−67.08	16	25	830	0.4	Agricultural	SS	Vertisol	U Puerto Rico
Ponce	17.99	−66.64	1	27	901	5.0	Savanna	O	Mollisol	Municipality of Ponce, PR
Steigerwaldt	45.51	−89.58	477	5	809	5.5	Temperate deciduous forest	SO	Spodosol	Steigerwaldt Land Services
Tree Haven	45.49	−89.59	461	5	809	23.0	Temperate deciduous forest	CL	Spodosol	University of Wisconsin Stevens Point
UNDERC	46.23	−89.54	520	5	751	24.0	Temperate deciduous forest	CL	Spodosol	U Notre Dame
Konza - Core	39.10	−96.56	415	13	835	1.5	Grassland	SS	Mollisol	Nature Conservancy/Kansas State U
Konza - Relocatable	39.11	−96.61	323	13	835	1.5	Agricultural	SS	Mollisol	Nature Conservancy/Kansas State U
U Kansas Bio Station	39.04	−95.19	321	14	940	19.0	Temperate deciduous forest	CL	Inceptisol	U Kansas
Oak Ridge Nat'l Lab	35.96	−84.28	342	15	1352	28.0	Temperate deciduous forest	CL	Ultisol	US DOE
Great Smokey Mtns NP	35.69	−83.50	661	13	1453	30.0	Temperate deciduous forest	CL	Ultisol	*US NPS
Mountain Lake Res. Stn.	37.38	−80.52	1170	8	1131	18.0	Temperate deciduous forest	CL	Entisol	U Virginia
Talladega Nat'l Forest	32.95	−87.39	164	15	1384	25.0	Temperate coniferous forest	SO	Ultisol	USDA FS
Choctaw NWA	31.85	−88.17	11	18	1524	35.0	Temperate deciduous forest	CL	Inceptisol	DOI NWA
Dead Lake WMA	32.54	−87.80	24	17	1415	30.0	Temperate deciduous forest	CL	Ultisol	US Army Corps of Engineers
Dakota-Coteau	47.16	−99.11	575	4	478	0.4	Grassland	SS	Mollisol	State of North Dakota Land Trust
Northern Great Plains Res. Lab	46.77	−100.92	589	5	434	0.4	Agricultural	SS	Mollisol	USGS/DOI FWS
Woodworth	47.13	−99.24	590	4	478	1.0	Agricultural	SS	Mollisol	USGS/DOI FWS
Central Plains Exp. Range	40.82	−104.75	1653	9	322	0.4	Grassland	SS	Mollisol	USDA ARS
Sterling	40.46	−103.03	1365	10	422	1.0	Agricultural	SS	Mollisol	Private,
Rocky Mtn. NP (Castnet)	40.28	−105.55	2742	7	998	19.0	Temperate coniferous forest	SO	-	DOI NPS
LBJ Nat'l grasslands	33.40	−97.57	272	18	864	13.0	Temperate deciduous forest	SO	Alfisol	USDA FS
M. Klemme Range Res. Station	35.41	−99.06	520	15	780	1.0	Agricultural	SS	Inceptisol	Oklahoma State U, Oklahoma Agricultural Experimental Station
Northcutt	33.89	−96.84	212	17	970	4.0	Savanna	O	Inceptisol	Private,
Bozeman	45.66	−111.05	1503	7	491	1.0	Agricultural	SS	Mollisol	Montana State U
Loch Leven	45.46	−110.62	1448	6	432	0.4	Grassland	SS	Mollisol	Montana Dept of Fish, Wildlife and Parks
Yellowstone NP	44.95	−110.54	2129	7	249	14.0	Temperate coniferous forest	SO	-	*DOI NPS
Niwot Ridge	40.05	−105.58	3478	−4	930	0.2	Tundra	SS	Inceptisol	USDA FS
Fraser Exp. Forest	39.86	−105.86	3526	1	584	14.0	Boreal/montane coniferous forest	SO	Inceptisol	USDA FS
Moab	38.25	−109.39	1800	14	239	0.2	Grassland	SS	Aridisol	BLM
Santa Rita Exp. Range	31.91	−110.84	999	22	303	2.0	Shrubland	SS	Entisol	U Arizona
Jornada Exp. Range	32.59	−106.84	1321	18	241	0.4	Grassland	SS	Aridisol	USDA ARS
Onaqui-Benmore	40.18	−112.45	1654	9	274	1.2	Shrubland	SS	Aridisol	BLM/USDA FS
Murray	40.65	−111.92	1302	12	450	1.0	Grassland	SS	- (anthropodisol)	City of Murray, UT
Red Butte	40.78	−111.80	1676	12	450	10.0	Temperate deciduous forest	CL	Mollisol	USDA FS/U Utah
Wind River RNA	45.82	−121.95	368	9	2223	50.0	Temperate coniferous forest	CL	Andisol	USDA FS
Abby Road	45.76	−122.33	367	9	2249	1.0	Temperate coniferous forest	SS	Andisol	State of Washington Dept. Natural Resources
Thayer	45.71	−122.34	557	9	2249	12.0	Temperate coniferous forest	CL	Inceptisol	State of Washington Dept. Natural Resources
Thyme	45.71	−122.38	301	9	2249	1.0	Temperate coniferous forest	SS	Ultisol	State of Washington Dept. Natural Resources
Good Seed	45.78	−122.30	559	9	2249	1.0	Temperate coniferous forest	SS	Ultisol	State of Washington Dept. Natural Resources
Soaproot Saddle	37.03	−119.26	1210	13	957	32.0	Temperate coniferous forest	SO	Alfisol	USDA FS
Lower Teakettle	37.01	−119.01	2149	8	957	35.0	Temperate coniferous forest	SO	Entisol-Inceptisol	USDA FS
San Joaquin Exp. Range	37.11	−119.73	397	17	375	21.0	Savanna	O	Alfisol-Inceptisol	USDA FS
Toolik Lake LTER	68.66	−149.37	827	−9	316	0.3	Tundra	SS	Gelisol	BLM
Pump Station 2	69.60	−148.67	142	−9	316	0.3	Tundra	v	Gelisol	BLM
Barrow Exp. Observatory	71.28	−156.62	7	−12	105	0.3	Tundra	SS	Gelisol	*Ukpeaġvik Iņupiat Corporation*
Caribou-Poker	65.15	−147.50	239	−3	262	8.0	Boreal/montane coniferous forest	SO	Inceptisol	UAF
Poker Flats	65.11	−147.42	472	−3	262	8.0	Boreal/montane coniferous forest	SS	Inceptisol	Alaska Dept Natural Resource/UAF
Eight Mile Lake	63.88	−149.22	684	−2	366	0.3	Tundra	SS	Gelisol	Alaska Dept Natural Resource
Delta Junction	63.88	−145.75	504	−3	305	10.0	Boreal/montane coniferous forest	SO	Inceptisol	BLM
Kenai NWA	60.53	−150.58	110	1	483	12.0	Boreal/montane coniferous forest	CL	Spodosol	DOI NWA

All sites were given access to measure soil properties by the owner, no soil samples were taken, and (*) denotes that a research permit was needed to gain access to the site.

Note: - = soil order unknown, USDA = US Department of Agriculture, FS = Forest Service, ARS = Agricultural Research Service, DOI = US Department of Interior, BLM = US Bureau of Land Management, DOE = US Department of Energy, UAF = University of Alaska, Fairbanks, FWS = US Fish and Wildlife Service, USGA = US Geological Survey, NPS = National Park Service, LTER = long term ecological research site, NWA = National Wildlife Area, RNA = Research Natural Area, WMA = Wildlife Management Area, NP = National Park, BS = Biological Station.

Soil taxonomic information at a site was gathered from the USDA Natural Resource Conservation Service's Web Soil Survey, except for Wind River [48], Bartlett Experimental Forest (www.fs.fed.us/ne/durham/4155/bartlett.htm#SOI), and Eight Mile Lake [49]. Soil taxonomic information was not available for Yellowstone National Park, WY, or Rocky Mountain National Park, CO, while the soil at Murray, UT, was classified by the Web Soil Survey as *dumps* (*i.e.*, anthropogenosol), which cannot be assigned to a specific soil order. Mean annual temperature and mean annual total precipitation data were gathered from local site sources when available, and from climate data for nearby towns (www.usclimatedata.com) when site-specific information was unavailable.

Field-based soil temperature and moisture data were collected over 1 to 2 days at each site between August 2009 and April 2011 (with most sites visited between March and October 2010) and were timed to coincide with peak growing season. The only exception was sampling at Mountain Lake, VA, which occurred prior to budburst due to logistical constraints. Our measurements corresponded to the growing season because this is the time of year when most biological activity occurs. It should be noted that spatial variability of soil properties can change seasonally [44], *i.e.*, there is often intra-annual nonstationarity in both functional linkages and spatial correlation), although this does not always occur [12], [21], [23], [26], [50]. As such, the spatial variability that we observed may not reflect spatial variation among other seasons or years.

Soil temperature, T_s_ (°C), was measured at 0–12 cm depth with platinum resistance temperature sensors (RTD 810, Omega Engineering Inc., Stamford CT) and soil water content, SWC (vol H_2_O vol^−1^ soil expressed as a percentage) was measured at a 0–15 cm depth with time domain dielectric sensors (CS616, Campbell Scientific Inc., Logan UT). Due to the large number of sites and soil types it was not possible to calibrate the soil moisture sensor to the soil type at each site; instead a manufacturer recommended equation was used to convert the sensor measurement of the dielectric constant to SWC [51], [52]. The sensors were inserted into the soil and allowed to stabilize prior to data acquisition. Data at each measurement point were acquired at 1 s (execution interval), and descriptive statistics were calculated over 30 s averaging periods with a datalogger (CR3000, Campbell Scientific Inc.). The sampling design was similar among the sites, but it was not identical due to site-specific space constraints (*e.g.*, property boundaries) and obstacles (*e.g.*, rock outcrops, roads, streams, tree trunks, and stone walls). The measurements were taken along 2 to 4 intersecting transects at each site, with the number and length of transects depending on site topography and property size. The intersection point of the transects was typically at the center of each transect. The length of transects ranged from 42 m to 210 m. The starting, center and ending points of each transect were recorded by a GPS unit, which was used to calculate distances during semivariogram analysis.

The determination of sampling locations followed the cyclic sampling logic used by Bond-Lamberty *et al.* [23], with measurements taken at 0, 2, 8, 28, 38, and 42 m (noted as transect points) and with this spacing scheme repeated until the end of the transect was reached (*i.e.*, the next sampling points would occur at 44, 50, 70, 80, 84, 86, 92 … m). In addition, two more T_s_ and SWC measurements were made at −0.3 m and +0.3 m from each transect point along the axis of the transect, thus resulting in a total of 3 T_s_ and 3 SWC measurements made at each transect point (Figure S1). The minimum and maximum distance between measurements ranged from 0.3 m to at least 84 m at each site, and up to 210 m at some sites, as a result, we were able to capture both small spatial scale (∼0.3 m) and the plot-to-footprint scale (∼100's m^−2^) variability. Measurements were taken at 134±1 (mean ±1 standard error, convention used throughout the text, unless otherwise noted) locations per site (maximum = 156, minimum = 111). The number of measurement points at each site exceeded the recommendation of at least 100 points to construct representative isotropic (*i.e.*, non-directional) semivariograms with soil data [53], [54].

It typically took ∼2.5 to 5 hours to collect the data at each site, during which time natural changes to T_s_ and SWC could occur, *e.g.*, solar heating of the soil, evaporation and/or transpiration. Thus, we also continuously collected T_s_ and SWC with a second, stationary suite of 3 pairs of identical T_s_ and SWC sensors (spaced 0.3 m apart) adjacent to the point where all the transects intersected to estimate diurnal and other weather-related influences on the measurements. Data at were acquired at 1 s (execution interval), and descriptive statistics were calculated over 30 sec averaging periods with a datalogger (Models CR1000, Campbell Scientific Inc.). T_s_ and SWC data from the transects and the stationary system, as well as measurement coordinates, that were used to create the semivariograms are available available by completing the request form at the bottom of the webpage: http://neoninc.org/pds/FIU007.php [55].

### Semivariograms and model fitting

Two primary approaches were used to de-trend temporal changes from the data collected along the transects, such that the remaining variability in the data can be attributed to its spatial sources and quantified with a semivariogram. First, mean T_s_ and SWC values from the stationary location were subtracted from transect data at each corresponding time of day to produce *corrected* data. Second, a linear regression based on the relationship between time of day and the *corrected* T_s_ and SWC data was fitted, and the residuals calculated. These residuals were then used to construct the semivariogram. In the context of geostatistics, de-trending is driven by the need to satisfy the second-order stationarity requirement, which needs to be met in order to ensure that the resulting covariance function is valid, *e.g.*, [24]. At 5 sites (Sterling, Rocky Mountain NP, Central Plains Exp. Range, Caribou Poker, and Northcutt) the stationary system was either unavailable or malfunctioned. Therefore, de-trending was done using the linear regression approach only. If additional patterns (*i.e.*, gradients in T_s_ or SWC across the sampling site or non-normal distribution) were still visible in the data after removing temporal trends, we used a third method to de-trend the data based on topographic relief (elevation, aspect, and slope calculated from a digital elevation map) to meet the assumptions of the semivariogram model. This third method consisted of adding elevation, aspect, and slope in all combinations (*i.e.*, elevation×aspect×slope, elevation×aspect, elevation×slope, etc.) to the time of day linear regression described above. Linear regressions that were not significant (p>0.05) were excluded, and the residuals were recalculated. De-trending using elevation, slope, and/or aspect was necessary at 18 sites (Barrow Environmental Observatory, Poker Flats, Eight Mile Lake, Woodworth, Northern Great Plains Research Lab, Tree Haven, Steigerwaldt, Upper Teakettle, Konza – Core, UK Biological Station, Harvard Forest, Niwot Ridge, Smithsonian Conservation Biology Institute, Ordway-Swisher BS, Great Smoky NP, Talladega Nat'l Forest, Choctaw WMA, and Murray).

Data collected were used for geospatial analyses in the R statistical computing language with the geoR package [56], [57]. At each site the empirical semivariogram, *ŷ*(h), which is half the average squared difference between data pairs, was calculated using the following equation,
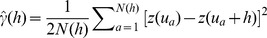
(1)where, *z*(u*_α_*), *α* = 1, 2, …, *n* denotes the set of T_s_ or SWC data, u*_α_* is the vector of spatial coordinates of the *α*_th_ observation, *h* represents a distance separating pairs of data, and *N*(h) is the number of data pairs separated by a given distance. In general, the covariance function for any pair of data that is *h* units apart can be represented as,
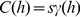
(2)where, *s* is a variance parameter and *γ(h)* is any positive definite correlation function. The correlation function used in this work was a spherical model with the following form,
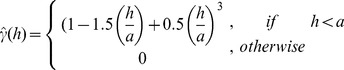
(3)where, *a* is the semivariogram range. In addition, we allowed for a nugget term, which is simply added to the covariance function. Approximate values for the range, sill, and nugget were estimated from the experimental semivariogram to seed the model and best-fit values were subsequently estimated using weighted least squares. It is important to note that a key goal of this study is to provide meaningful comparisons of the results across a number of sites, and this comparability was weighed as more important than a potentially negligible difference in the fit of a model for varying functional forms of the variogram, i.e., same sources of uncertainty in the model fit for all sites. Based on these two constraints, we selected the functional form of the variograms (spherical) that provided the best fit at most of the sites and applied it to all sites for the sake of consistency.

Lag spacing was set to 1 m for every empirical semivariogram. Preliminary tests with T_s_ and SWC data from Eight Mile Lake, AK, and Klemme, OK, showed that changing the lag spacing to 1, 2, or 5 m did not alter the estimated range value in any systematic manner, and the change was ±9% on average, which corresponded to ±4 m. Similarly, the coefficient of variation estimated from the semivariogram model was strongly positively correlated with the coefficient of variation calculated via the traditional method (see *Coefficient of variation and sample size* subsection in the *Results* section), indicating that 1 m lag spacing allowed the model to accurately describe the variability in the dataset.

### Calculating CVs and sample size

The coefficient of variation for T_s_ and SWC was calculated for each site in two ways: 1) the traditional method of dividing the standard deviation by the mean (referred to as CV_Traditional_); and 2) using the sill to estimate standard deviation and dividing that value by the mean (referred to as CV_Sill_) as follows,
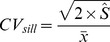
(4)where, 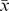 represents the sample mean, and 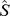 represent the semivariogram sill.

We calculated the sample size (*n*) needed to estimate the mean T_s_ and SWC to within 10% of the spatial mean and with 90% confidence using [4],
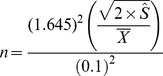
(5)where, 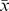 represents the sample mean, and 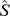 represents the semivariogram sill. The constants 0.1 and 1.645 represent the accuracy requirement (*i.e.*, 10%) expressed as a proportion and the value of *t* statistic that corresponds to the 90% confidence interval, respectively. These accuracy and confidence thresholds were chosen because they are conservative enough to meet the goals of most studies. An assumption of the hypothesis test underlying this analysis is that the data are normally distributed, which we ensured prior to constructing the semivariograms.

#### Statistical analyses

Several of the hypotheses that we tested involved relating site characteristics to dependent variables. Some site characteristics (*i.e.*, factors in the statistical tests) were continuous variables (latitude, longitude, mean annual air temperature, mean annual precipitation, elevation, canopy height, mean T_s_, and mean SWC), while others were categorical variables (ecosystem type, canopy structure, and soil type). Some levels of the categorical site characteristics contained too few sites to allow statistical analyses to be conducted. For example, Andisols were only found at 2 sites (Wind River and Abby Road, WA) and a tropical deciduous forest ecosystem only occurred at 1 site (Guanica, PR). As a result, levels were combined to increase the number of sites within each level prior to conducting the statistical tests. Ecosystem types were grouped into 6 levels: deciduous forests (both tropical and temperate), temperate coniferous forests, grasslands (incl. shrublands, and savannas), agricultural, boreal (including montane forests), and tundra. While we recognize that short stature ecosystems (*e.g.*, grasslands) can have closed canopies with only small gaps between neighboring plants or open canopies with substantial amounts of bare ground between plants, they clearly differ in structure from taller stature ecosystems. The ecosystem and canopy structure classifications were somewhat subjective and some sites could have been reasonably placed in two or more different groups, but the chosen category represents our best judgment. Canopy structure was grouped into 3 levels: closed canopy ecosystems, open and semi-open canopy ecosystems, and short stature ecosystems. Vegetation canopy heights were measured in the field and reflect the average height of the tallest component of the plant community (*e.g.*, in a savanna ecosystem the canopy height reflects the average height of the trees, rather than the grasses). Soils were grouped according to soil orders: Inceptisols, Spodosols, Ultisols, Mollisols, Gelisols, and other (incl. Vertisols, Andisols, Alfisols, Entisols, Aridisols, as well as sites with >1 soil order and sites where the soil order was unknown).

In all statistical models described below, factors in the model that had a p value of >0.1 were removed in a stepwise manner and the model was re-run until all remaining factors had a p value of <0.1. Statistical significance was assumed at p<0.05 for all tests. Post-hoc Tukey HSD tests were performed when ANOVAs identified a significant effect of the categorical factor(s).

We expected that many of the site characteristics would covary (*e.g.*, latitude and mean annual air temperature) and it is necessary to understand this covariation when interpreting the statistical tests that we performed. To assess covariation in the explanatory variables used in the statistical tests, we calculated correlation coefficients for each combination of site characteristics (latitude, longitude, mean annual air temperature, mean annual precipitation, elevation, canopy height, mean T_s_, and mean SWC). Categorical site characteristic factors (*i.e.*, ecosystem type, canopy structure, and soil type) were excluded from this analysis. Stepwise removal of non-significant factors from statistical tests tends to remove factors that covary with one-another, therefore, interpretation of a significant effect caused by a site characteristic should also consider other covarying site characteristics.

At some sites it was not possible to estimate the range or sill because the semivariogram did not reach an asymptote (see *Semivariograms* subsection in the *Results* section); therefore these sites were excluded from further statistical analysis. We tested whether these sites had different characteristics than sites where the semivariograms did reach an asymptote to determine whether this could influence the interpretation of our results. To achieve this, nominal logistic models were performed in JMP (SAS Institute Inc., Cary, NC) to determine whether significant differences in latitude, longitude, elevation, mean annual air temperature, mean annual precipitation, canopy height, mean T_s_, mean SWC, and maximum semivariogram lag distance existed between sites where semivariograms did and did not reach an asymptote. Categorical site characteristic (*i.e.*, ecosystem type, canopy structure, and soil type) were excluded from this analysis due to the limited number of sites within each level of the categories.

ANOVAs were performed in JMP to determine the relationship between site characteristics and T_s_ and SWC. The factors in the ANOVAs were latitude, longitude, elevation, mean annual air temperature, mean annual precipitation, canopy height, ecosystem type, canopy structure, and soil type and the dependent variables were mean T_s_ and mean SWC. Both mean T_s_ and mean SWC were log_10_ transformed to meet assumptions of normality and homogeneity.

ANOVAs were also used to test hypotheses 1–4. ANOVAs were performed in JMP to assess the relationship between site characteristics and semivariogram range, nugget, and sill, as well as estimated sample size required to meet the accuracy requirement for T_s_ and SWC. Semivariogram nugget data for T_s_ and SWC met the assumptions of normality and homogeneity; however, range, sill, and sample size data required log_10_ transformations to meet these assumptions.

Because some factors often explained the majority of variation in an ANOVA, relatively weak relationships that were statistically significant were sometimes masked by the dominant factors when plotted as graphs. This is because the ANOVA accounts for variability in the data caused by others factors, whereas the graphs, which are based on raw data, do not. This should be taken into account when comparing the statistical results to the graphs.

A Spearman's rank correlation was performed in JMP to assess the relationships between mean T_s_ and mean SWC to determine whether they were significantly correlated. Additional Spearman's rank correlations were performed in JMP to assess the relationships among T_s_ range and SWC range, T_s_ nugget and SWC nugget, T_s_ sill and SWC sill, and T_s_ sample size and SWC sample size (Hypothesis 5); and T_s_ range, nugget, and sill, as well as SWC range, nugget, and sill (Hypothesis 6).

Paired one sample *t*-tests (JMP) were used to assess whether range, nugget, sill, and sample size values differed between T_s_ and SWC among the sites to test Hypothesis 6. In addition, to test whether the semivariograms accurately described the variability in T_s_ and SWC at each site, linear regressions (JMP) were used to assess the relationship between CV_Traditional_ and CV_Sill_ for both T_s_ and SWC.

The range and sample size for T_s_ and SWC were plotted against the cumulative proportion of sites to investigate the frequency distribution. These relationships were modeled with a 3-parameter logistic curve using SigmaPlot (Systat Software Inc., Chicago, Illinois).

In the interest of brevity, we only present graphs relating to a subset of site characteristics in this paper, namely ecosystem type, canopy structure, and soil order. The relationship between dependent variables and other site characteristics, including latitude, longitude, elevation, mean annual air temperature, mean annual precipitation, mean T_s_, and mean SWC, are presented in the Appendices.

## Results

### Site characteristics

**Figure 1:**
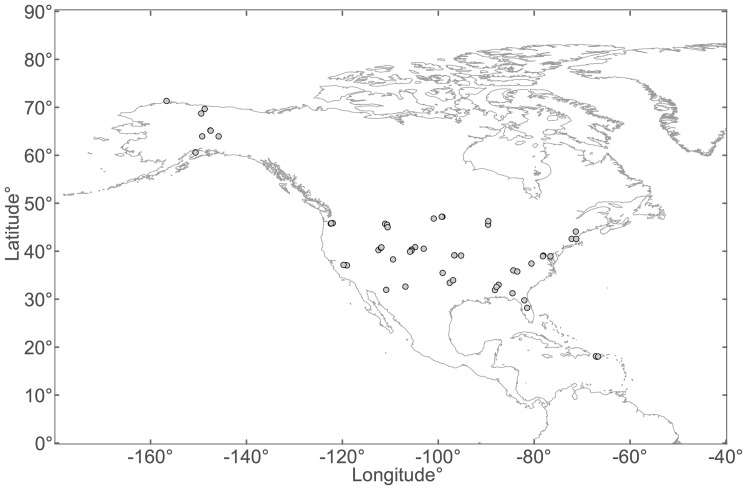
The sampling sites (open circles) were widely distributed throughout the US.

**Figure 2:**
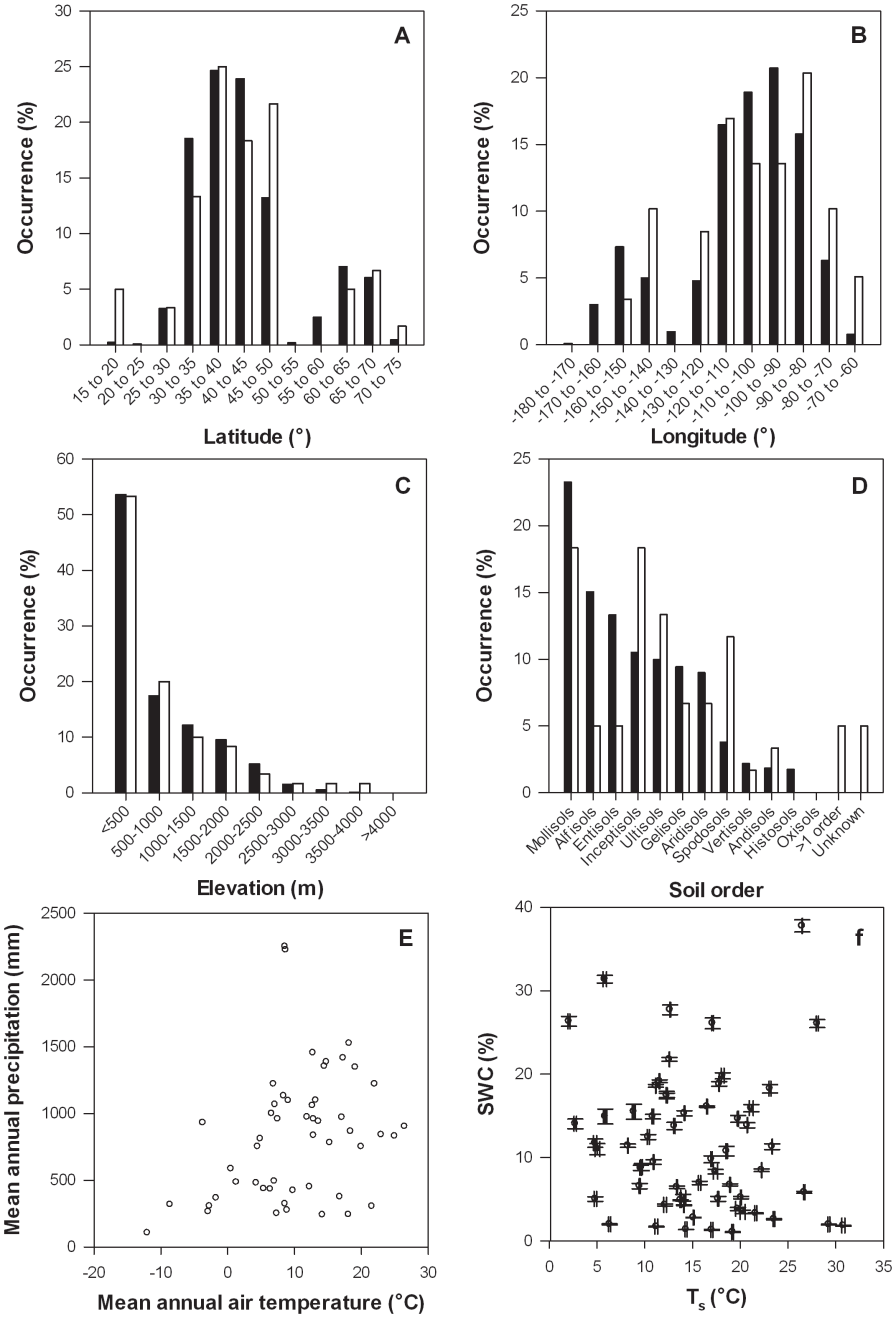
The sites were broadly representative of the US. Percent occurrence of land in latitudinal (a), longitudinal (b), elevational (c), and soil order (d) categories in the US (black bars), and our study sites (white bars). The study sites also occupied a wide range of climates (e) and T_s_ and SWC conditions at the time of samplings (f). Error bars in (f) represent ±1 SE.

The sites encompassed a continental-scale range of eco-climatic properties (Table 1, Figure 1), and were broadly representative of the proportion of US land in different latitudinal, longitudinal, and elevational categories, as well as spanning a wide range of soil types, climate space, and soil temperature and moisture conditions at the time of sampling (Figure 2). T_s_ varied from 2.1±0.1°C (mean ±1SE) at Pump Station 2, AK, to 30.8±0.2°C at Santa Rita Exp. Range, AZ, while SWC varied from 1.1±0.1% at Burlington, MA, to 37.8±0.7% at Ponce, PR (Table 2). T_s_ and SWC estimates were not correlated with each other among the sites (p = 0.143), resulting from several sites with contrasting T_s_ and SWC combinations (*i.e.*, warm and wet, warm and dry, cool and wet, and cool and dry, Figure 2f).

**Table 2:** Summary statistics of T_s_ and SWC measurements, as well as range, sill, and nugget estimates based on the semivariogram model, at each site.

	T_s_										SWC										Sampling date‡
Site	Mean (°C)	SD	CV_Traditional_	Maximum lag	Range (m)*	Nugget (% of sill)*	Sill*	CV_Sill_	Sample size†	n	Mean (%)	SD	CV_Traditional_	Maximum lag	Range (m)*	Nugget (% of sill)*	Sill*	CV_Sill_	Sample size†	n	
Harvard Forest	17.7	0.6	0.04	100	74	55	0.46	0.05	0.8	144	5.0	3.5	0.69	100	17	62	9.60	0.87	203.8	144	7/8/2010
Burlington	19.2	0.8	0.04	100	9	56	0.67	0.06	1.0	119	1.1	0.6	0.52	100	10	31	0.44	0.88	209.5	120	7/7/2010
Bartlett Exp. Forest	17.0	0.9	0.05	100	40	67	0.98	0.08	1.8	126	9.8	4.6	0.47	100	68	27	29.41	0.78	166.1	126	7/6/2010
Blandy Farm	22.2	0.4	0.02	100	-	-	-	-	-	129	8.5	1.8	0.21	100	47	53	3.56	0.31	26.8	129	8/18/2010
Smithsonian CRC (SCBI)	20.7	0.4	0.02	80	5	16	0.05	0.02	0.1	126	13.9	3.6	0.26	80	-	-	-	-	-	126	8/19/2010
Smithsonian ERC	23.3	0.4	0.02	80	34	15	0.16	0.02	0.2	123	11.3	3.9	0.34	80	32	41	13.40	0.46	56.7	123	8/17/2010
Ordway-Swisher BS	14.3	1.2	0.08	100	6	5	0.58	0.08	1.5	144	1.4	0.3	0.22	100	23	64	0.09	0.31	25.7	144	3/24/2010
J. Jones Ecological Research station	14.1	0.3	0.02	100	35	31	0.08	0.03	0.2	129	4.3	0.7	0.17	100	8	96	0.54	0.24	15.8	129	3/21/2010
Disney Wilderness Preserve	16.5	0.6	0.04	100	4	25	0.31	0.05	0.6	144	16.1	1.0	0.06	95	78	77	1.00	0.09	2.1	144	3/23/2010
Guanica State Forest	26.7	0.7	0.03	84	-	-	-	-	-	129	5.8	1.4	0.24	84	15	32	2.35	0.37	37.3	129	3/18–19/2010
Lajas Ag. Exp. St.	28.1	1.2	0.04	65	12	22	0.63	0.04	0.4	120	26.0	5.3	0.20	65	24	57	27.17	0.28	21.7	120	3/17–18/2010
Ponce	26.5	0.4	0.02	100	25	18	0.21	0.02	0.2	126	37.8	7.9	0.21	100	25	9	73.66	0.32	28.0	121	10/12/2010
Steigerwaldt	19.8	0.8	0.04	100	-	-	-	-	-	144	14.6	4.9	0.33	100	-	-	-	-	-	144	8/12/2010
Tree Haven	18.6	0.9	0.05	80	-	-	-	-	-	129	10.8	6.5	0.60	80	12	40	15.87	0.52	74.2	129	8/11/2010
UNDERC	18.9	0.4	0.02	100	70	68	0.13	0.03	0.2	141	6.7	1.6	0.23	100	20	75	2.70	0.35	32.4	141	8/10/2010
Konza - Core	14.2	0.2	0.02	100	18	58	0.04	0.02	0.1	129	15.3	3.6	0.23	100	52	75	13.70	0.34	31.7	129	5/12/2010
Konza - Relocatable	12.6	0.2	0.02	100	35	34	0.04	0.02	0.1	123	21.8	2.6	0.12	100	67	38	9.02	0.20	10.3	123	5/13/2010
U Kansas Bio Station	12.7	0.5	0.04	84	7	18	0.06	0.03	0.2	129	27.7	6.7	0.24	84	-	-	-	-	-	129	5/11/2010
Oak Ridge Nat'l Lab	13.4	0.4	0.03	84	30	16	0.18	0.04	0.5	129	6.4	1.7	0.26	84	-	-	-	-	-	129	4/19/2010
Great Smokey Mtns NP	11.2	0.3	0.02	80	70	54	0.05	0.03	0.2	129	18.6	1.9	0.10	80	-	-	-	-	-	129	4/21/2010
Mountain Lake Res. Stn.	8.2	0.4	0.05	100	79	42	0.11	0.06	0.9	129	11.4	2.2	0.19	100	70	70	5.55	0.29	23.1	129	4/22/2010
Talladega Nat'l Forest	20.2	3.0	0.15	100	94	36	4.78	0.15	6.3	129	3.5	2.5	0.72	100	20	20	5.63	0.97	252.9	129	4/16/2010
Choctaw NWA	17.1	1.1	0.06	84	16	8	0.63	0.07	1.2	129	26.1	7.4	0.28	84	45	42	51.93	0.39	41.3	129	4/14/2010
Dead Lake WMA	17.8	0.3	0.02	80	-	-	-	-	-	132	18.8	3.1	0.17	80	-	-	-	-		132	4/19/2011
Dakota-Coteau	12.3	0.8	0.07	100	10	19	0.43	0.08	1.5	144	17.4	3.2	0.19	100	19	60	8.06	0.23	14.4	144	5/26/2010
Northern Great Plains Res. Lab	11.6	0.5	0.04	100	8	23	0.12	0.04	0.5	144	19.1	2.0	0.11	100	4	63	4.37	0.15	6.5	144	5/25/2010
Woodworth	15.7	1.3	0.08	100	14	65	0.89	0.09	2.0	144	6.9	2.4	0.35	100	8	53	2.89	0.35	32.5	144	5/27/2010
Central Plains Exp. Range	23.5	1.0	0.04	100	95	32	0.54	0.04	0.5	144	2.6	0.8	0.32	100	29	40	0.72	0.46	57.8	144	8/13/2009
Sterling	23.1	1.3	0.06	100	50	57	0.60	0.05	0.6	156	18.2	6.5	0.36	100	2	0	49.25	0.54	80.1	156	8/10/2009
Rocky Mtn. NP (Castnet)	11.2	1.7	0.15	84	-	-	-	-	-	132	1.7	0.5	0.32	84	12	42	0.31	0.46	57.3	132	8/18–19/2009
LBJ Nat'l grasslands	20.1	0.6	0.03	100	9	2	0.48	0.05	0.6	129	5.2	1.5	0.29	100	3	17	2.49	0.43	50.0	129	9/28/2010
M. Klemme Range Res. Station	18.1	1.6	0.09	100	22	11	2.31	0.12	3.8	129	19.8	4.1	0.21	100	72	23	21.98	0.33	30.4	128	4/30/2010
Northcutt	19.7	2.8	0.14	100	11	27	2.49	0.11	3.5	141	3.8	2.3	0.59	100	78	15	7.59	1.02	280.8	141	4/28/2010
Bozeman	21.1	2.3	0.11	100	25	7	3.47	0.12	4.2	123	15.9	4.8	0.30	100	23	49	25.99	0.45	55.6	123	7/20/2010
Loch Leven	21.6	1.4	0.06	100	15	44	0.82	0.06	0.9	138	3.3	0.6	0.19	100	70	36	0.57	0.32	27.9	138	7/21/2010
Yellowstone NP	12.1	1.7	0.14	100	31	13	3.02	0.20	11.1	132	4.3	1.4	0.33	100	15	28	2.36	0.51	69.0	132	7/22/2010
Niwot Ridge	8.8	1.7	0.20	80	5	3	2.60	0.26	18.0	123	15.5	10.0	0.65	84	27	9	83.27	0.83	187.9	123	7/30/2010
Fraser Exp. Forest	6.3	1.2	0.19	84	21	23	1.00	0.22	13.5	132	2.0	0.7	0.33	80	15	94	0.44	0.47	60.3	132	9/15/2010
Moab	15.1	0.5	0.04	100	16	64	0.12	0.03	0.3	144	2.8	0.4	0.16	100	37	58	0.22	0.24	15.6	144	10/6/2010
Santa Rita Exp. Range	30.8	1.9	0.06	100	2	0	0.79	0.04	0.5	141	1.8	0.5	0.30	100	17	38	0.32	0.44	52.7	141	8/25/2010
Jornada Exp. Range	29.3	1.2	0.04	100	46	73	0.57	0.04	0.4	141	2.0	0.5	0.26	100	-	-	-	-	-	141	8/23/2010
Onaqui-Benmore	13.8	0.8	0.06	84	14	12	0.61	0.08	1.7	135	4.9	0.8	0.16	84	-	-	-	-	-	135	5/18/2010
Murray	17.4	2.6	0.15	84	-	-	-	-	-	135	8.3	3.1	0.37	84	2	0	6.86	0.45	54.0	135	5/19/2010
Red Butte	10.8	1.0	0.09	65	22	42	0.81	0.12	3.7	132	14.9	3.2	0.21	65	15	79	10.44	0.31	25.4	132	5/20/2010
Wind River RNA	10.9	0.4	0.03	100	4	31	0.09	0.04	0.4	138	9.5	3.1	0.33	100	3	63	10.27	0.48	62.1	138	6/8/2010
Abby Road	9.6	1.0	0.11	100	3	30	1.02	0.15	6.0	141	8.8	3.7	0.42	100	21	44	16.15	0.65	114.1	141	10/20/2010
Thayer	9.5	0.6	0.06	70	40	58	0.42	0.10	2.5	111	6.6	3.4	0.51	70	-	-	-	-	-	111	10/21/2010
Thyme	12.3	0.6	0.05	100	17	32	0.40	0.07	1.4	129	17.5	5.1	0.29	100	27	53	27.81	0.43	49.3	129	6/10/2010
Good Seed	13.1	0.8	0.06	100	20	13	0.79	0.10	2.5	126	13.8	5.0	0.36	100	43	46	25.47	0.52	72.7	126	6/9/2010
Soaproot Saddle	17.0	1.1	0.07	84	9	13	1.18	0.09	2.2	129	1.3	0.5	0.36	84	3	18	0.24	0.52	73.5	129	7/15/2010
Lower Teakettle	14.0	2.9	0.20	80	11	2	6.75	0.26	18.6	129	5.2	4.4	0.86	80	13	2	20.06	1.22	404.6	129	7/15/2010
San Joaquin Exp. Range	9.7	1.2	0.12	100	6	0	0.96	0.14	5.6	144	8.8	5.0	0.57	100	8	6	19.91	0.71	137.8	144	2/22/2011
Toolik Lake LTER	2.7	1.6	0.59	100	3	40	2.65	0.85	194.6	144	14.0	6.9	0.49	100	5	58	52.77	0.73	145.1	144	6/19/2010
Pump Station 2	2.1	1.2	0.59	100	31	42	1.63	0.88	207.6	128	26.3	7.1	0.27	100	17	47	55.17	0.40	43.1	144	6/20/2010
Barrow Exp. Observatory	5.8	2.2	0.39	100	14	15	4.31	0.51	69.1	141	31.4	5.4	0.17	100	-	-	-	-	-	141	7/26/2010
Caribou-Poker	5.0	3.1	0.61	100	6	44	9.90	0.88	211.2	144	11.0	8.1	0.73	100	7	49	67.34	1.05	300.9	144	6/22/2010
Poker Flats	10.4	1.4	0.14	100	3	3	1.58	0.17	8.0	144	12.5	3.6	0.29	100	8	59	13.29	0.41	46.4	144	6/28/2010
Eight Mile Lake	5.9	2.1	0.36	80	72	81	4.65	0.52	73.5	138	14.9	10.6	0.71	80	14	74	94.74	0.92	230.4	138	6/29/2010
Delta Junction	4.8	1.9	0.40	100	44	31	4.28	0.61	100.4	138	11.7	5.8	0.49	100	6	34	33.23	0.70	131.2	138	6/23/2010
Kenai NWA	4.8	1.5	0.32	100	84	80	2.66	0.48	61.9	144	5.0	3.2	0.63	100	44	55	11.47	0.95	245.9	144	7/6/2010

The standard deviation (SD) is reported here because it was required to calculate CV_Traditional_.

- indicates that the semivariogram model did not reach its sill, which prevented sill, range, and nugget values from being accurately estimated.

Sample size required to estimate to with 10% of the spatial mean with 90% confidence.

Month/day/year format.

As expected, correlations demonstrated a substantial amount of covariation in the site characteristics (Table 3). For example, mean annual air temperature, mean T_s_, and longitude were all strongly negatively correlated with latitude. Weaker correlations were observed between mean annual precipitation and canopy height, and elevation and mean SWC. These correlations should be considered when interpreting the results, since the stepwise removal of non-significant factors from the ANOVA models tends to remove factors that covary from the model. For example, a significant relationship between longitude and a dependent variable would likely also be significant for latitude, mean annual air temperature, or mean T_s_ if longitude had not been included as a factor.

**Table 3:** Correlation coefficients indicating covariation among many site characteristics.

	Latitude	Longitude	Mean annual air temperature	Mean annual precipitation	Elevation	Canopy height	Mean T_s_	Mean SWC
Latitude	1	−0.808	−0.921	−0.288	−0.001	−0.211	−0.700	0.084
Longitude	−0.808	1	0.693	0.277	−0.173	0.309	0.654	−0.018
Mean annual air temperature	−0.921	0.693	1	0.284	−0.252	0.196	0.701	−0.061
Mean annual precipitation	−0.288	0.277	0.284	1	−0.271	0.412	0.005	0.025
Elevation	−0.001	−0.173	−0.252	−0.271	1	−0.154	−0.129	−0.325
Canopy height	−0.211	0.309	0.196	0.412	−0.154	1	0.012	−0.199
Mean T_s_	−0.700	0.654	0.701	0.005	−0.129	0.012	1	−0.115
Mean SWC	0.084	−0.018	−0.061	0.025	−0.325	−0.199	−0.115	1

**Figure 3:**
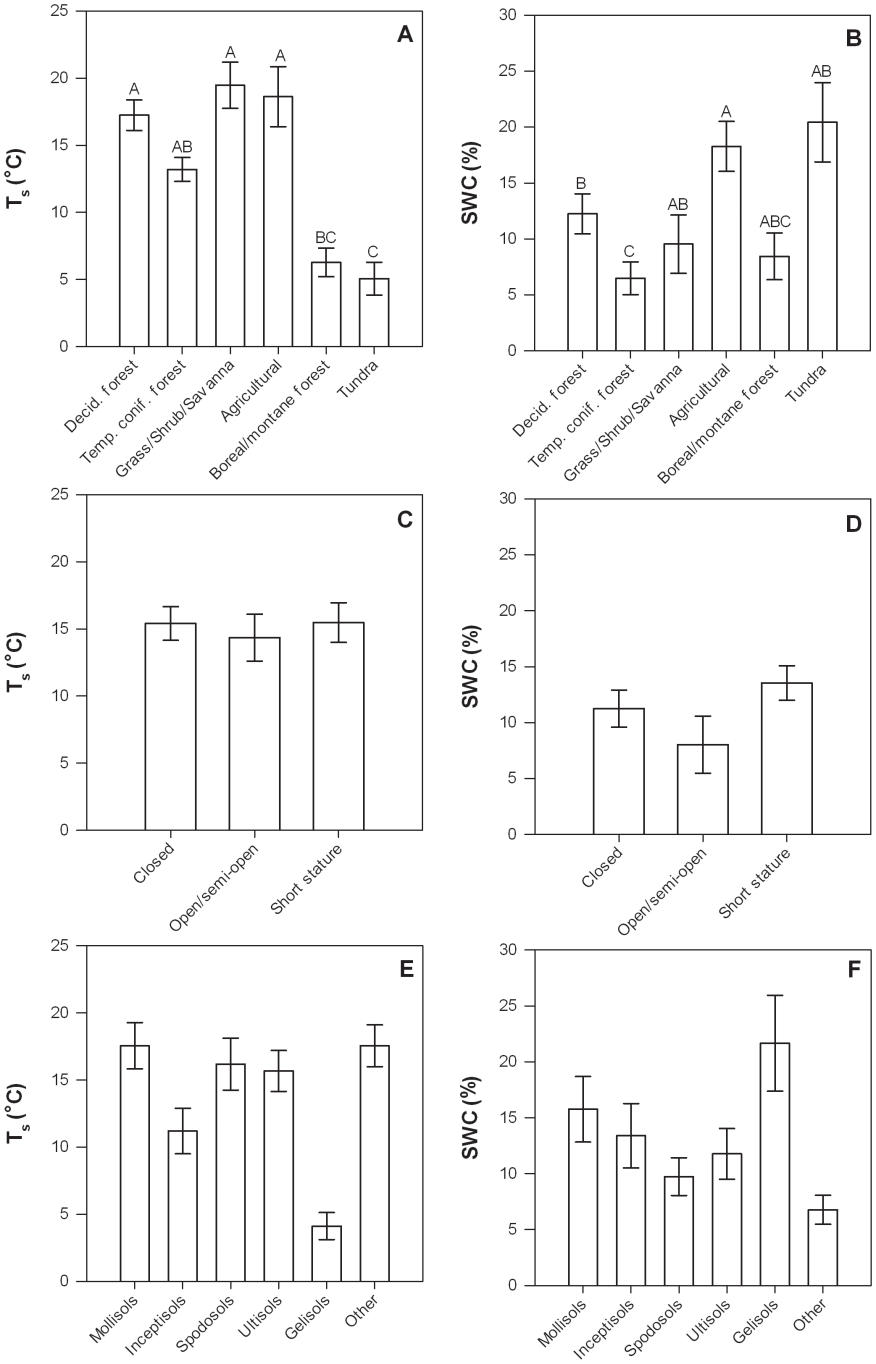
Mean T_s_ (left column) and SWC (right column) at each site in relation to ecosystem type (a, b), canopy structure (c, d), and soil type (e, f). Error bars represent ±1 SE. Bars with different letter were significantly different (p<0.05) based on Tukey HSD tests. The letter “A” corresponds to the largest least square mean(s) and subsequent letters (B, C, …) correspond to progressively smaller least square means.

Mean T_s_ among sites was negatively related to latitude and differed among ecosystems, *i.e.*, not surprisingly being higher in temperate ecosystems, and lower in montane and high-latitude ecosystems (Table 4, Figure 3). Mean T_s_ among sites followed expected relationships to mean annual air temperature, elevation, and soil type (*e.g.*, being lower in Gelisols, which are associated with cold climates; Figure 3), but the effects were not significant due to covariation among these factors and the factors that were statistically significant in the model (*i.e.*, latitude and ecosystem type). Mean SWC was positively related to mean total annual precipitation, decreased with increasing elevation and mean T_s_, and was significantly related to ecosystem type, being largest in tundra ecosystems, which have low evapotranspiration rates, and in agricultural ecosystems, several of which were irrigated, and the lowest in temperate coniferous forests (Table 4, Figure 3).

**Table 4:** Statistical significance (p values) of site characteristics on mean T_s_ and SWC, as well as semivariogram properties and derived values.

	T_s_					SWC				
	Mean	Range	Sill	Nugget	Sample size	Mean	Range	Sill	Nugget	Sample size
Latitude	<0.001	NS	NS	NS	NS	NS	NS	NS	NS	NS
Longitude	NS	NS	0.013	NS	0.003	NS	0.008	0.023	0.002	0.004
Elevation	NS	0.091	NS	NS	NS	0.032	NS	NS	NS	NS
Mean annual air temperature	NS	NS	0.003	NS	0.004	NS	NS	0.004	0.082	0.009
Mean annual precipitation	NS	NS	NS	NS	NS	0.007	NS	0.050	NS	0.002
Canopy height	NS	0.065	NS	0.024	0.087	NS	0.037	NS	NS	NS
Ecosystem type	<0.001	NS	NS	NS	NS	<0.001	NS	NS	0.015	0.071
Canopy structure	NS	0.019	<0.001	0.006	<0.001	NS	NS	0.053	<0.001	<0.001
Soil type	NS	0.009	NS	0.046	0.021	NS	NS	NS	0.026	NS
Mean T_s_	NA	NS	0.012	NS	NS	0.032	NS	0.046	0.001	0.004
Mean SWC	NS	NS	NS	0.005	NS	NA	NS	<0.001	0.072	0.037
										
Model r^2^	0.73	0.40	0.57	0.48	0.83	0.51	0.18	0.70	0.62	0.66

The r^2^ of the statistical model (observed versus predicted) is also shown.

NS: not significant (*i.e.*, p>0.1 and factor was removed from the statistical model), NA: not applicable.

### Semivariograms

In 12% and 17% of cases the semivariograms for T_s_ and SWC, respectively, had not reached an asymptote at the maximum lag distance used to construct the modeled semivariogram (Table 2). As a result, sill, range, and nugget (expressed as a percent of sill) values for these semivariograms were based on extrapolation, which may not accurately represent the *true* values. At 75% of sites both the T_s_ and SWC semivariogram reached an asymptote, at 13% of sites only the T_s_ semivariogram reached an asymptote, at 8% of sites only the SWC semivariogram reached an asymptote, and at the remaining 3% of sites no asymptote was reached for T_s_ or SWC. Sites with semivariograms that did not reach an asymptote were not different from other sites in terms of their environmental characteristics (*i.e.*, latitude, longitude, elevation, mean annual air temperature, mean annual precipitation, canopy height, mean soil temperature, or mean soil moisture). However, the SWC semivariograms that did not reach an asymptote were associated with shorter maximum lag distances (Table 5). Unless stated otherwise, all subsequent results are based solely on estimated sill, range, and nugget values from semivariograms that reached an asymptote. Since there were no significant differences in environmental characteristics between sites where the semivariogram did and did not reach an asymptote, there is no evidence that excluding sites where the semivariogram did not reach an asymptote introduced a systematic bias into the interpretation of our results.

**Table 5:** Site characteristics where the range value was larger than or less than the maximum lag distance covered by the semivariogram model, (mean ±1 SE). Range>maximum lag indicates that the semivariogram did not reach an asymptot.

	Latitude (°)	Longitude (°)	Elevation (m.a.s.l.)	Mean annual temperature (°C)	Mean annual precipitation (mm)	Canopy height (m)	Mean T_s_ (°C)	Mean SWC (%)	Maximum lag of semivariogram (m)	n
T_s_										
Range<maximum lag	43±2	−106±3	741±111	9±1	861±80	12±2	15±1	12±1	94±1	53
Range>maximum lag	37±4	−90±6	759±367	12±3	899±109	13±4	19±2	10±2	87±3	7
P value	NS	NS	NS	NS	NS	NS	0.098	NS	NS	
SWC										
Range<maximum lag	42±2	−105±3	778±122	9±1	841±76	11±2	15±1	11±1	95±1	50
Range>maximum lag	42±4	−102±8	572±168	10±3	990±211	16±4	15±2	14±3	86±3	10
P value	NS	NS	NS	NS	NS	NS	NS	NS	0.024	

NS: not significant (*i.e.*, p>0.1 and factor was removed from the statistical model).

### Semivariogram range

**Figure 4:**
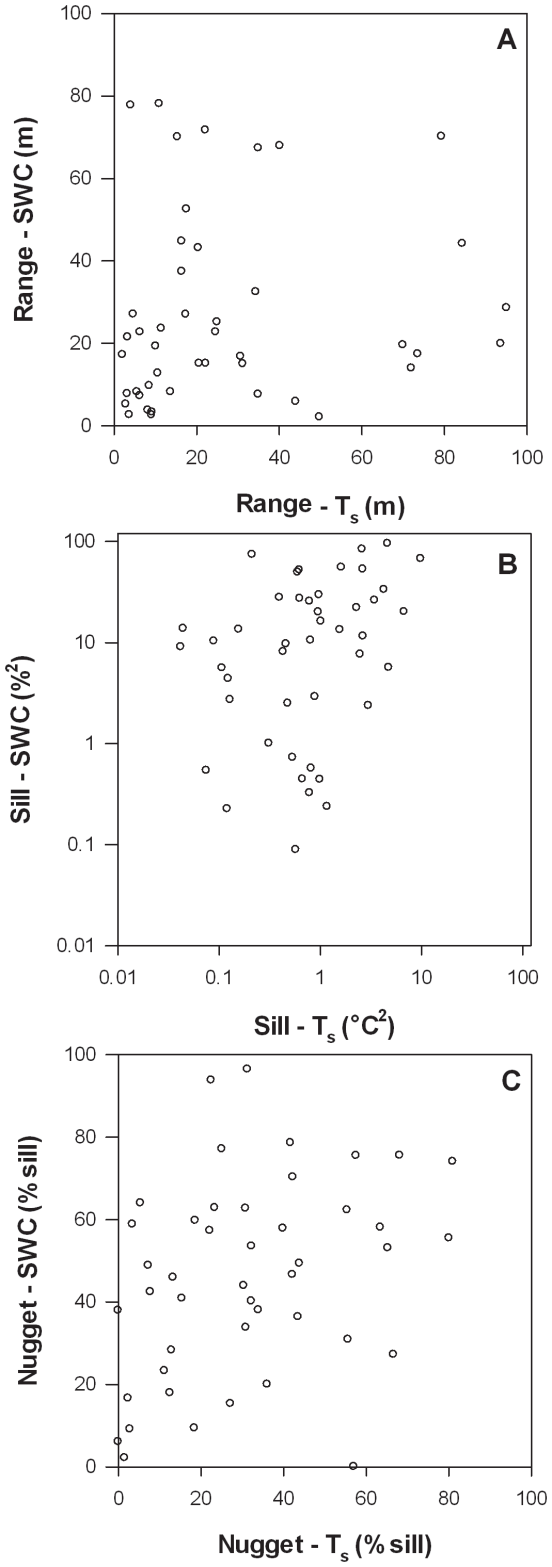
Semivariogram range (a), sill (b), and nugget (c) for T_s_ versus SWC at the sites. Sill values are plotted on a log scale due to large differences in magnitude across the sites.

The mean range value was 27±4 m and 26±3 m for T_s_ and SWC, respectively. A paired *t*-test demonstrated that T_s_ range values did not significantly differ from SWC range values (p = 0.773). Despite the similarity in the mean range, range values for T_s_ were not correlated with range values for SWC (Table 6; Figure 4a). At some sites, the T_s_ and SWC range values were similar, but at many sites one range value was substantially larger than the other.

**Table 6:** Correlation coefficients and statistical significance for T_s_ and SWC semivariogram properties and derived values.

	Spearman correlation coefficient	p value
T_s_ range – SWC range	0.26	0.089
T_s_ sill – SWC sill	0.36	0.016
T_s_ nugget – SWC nugget	0.34	0.021
T_s_ CV_Sill_ – SWC CV_Sill_	0.59	<0.001
T_s_ sample size – SWC sample size	0.59	<0.001

**Figure 5:**
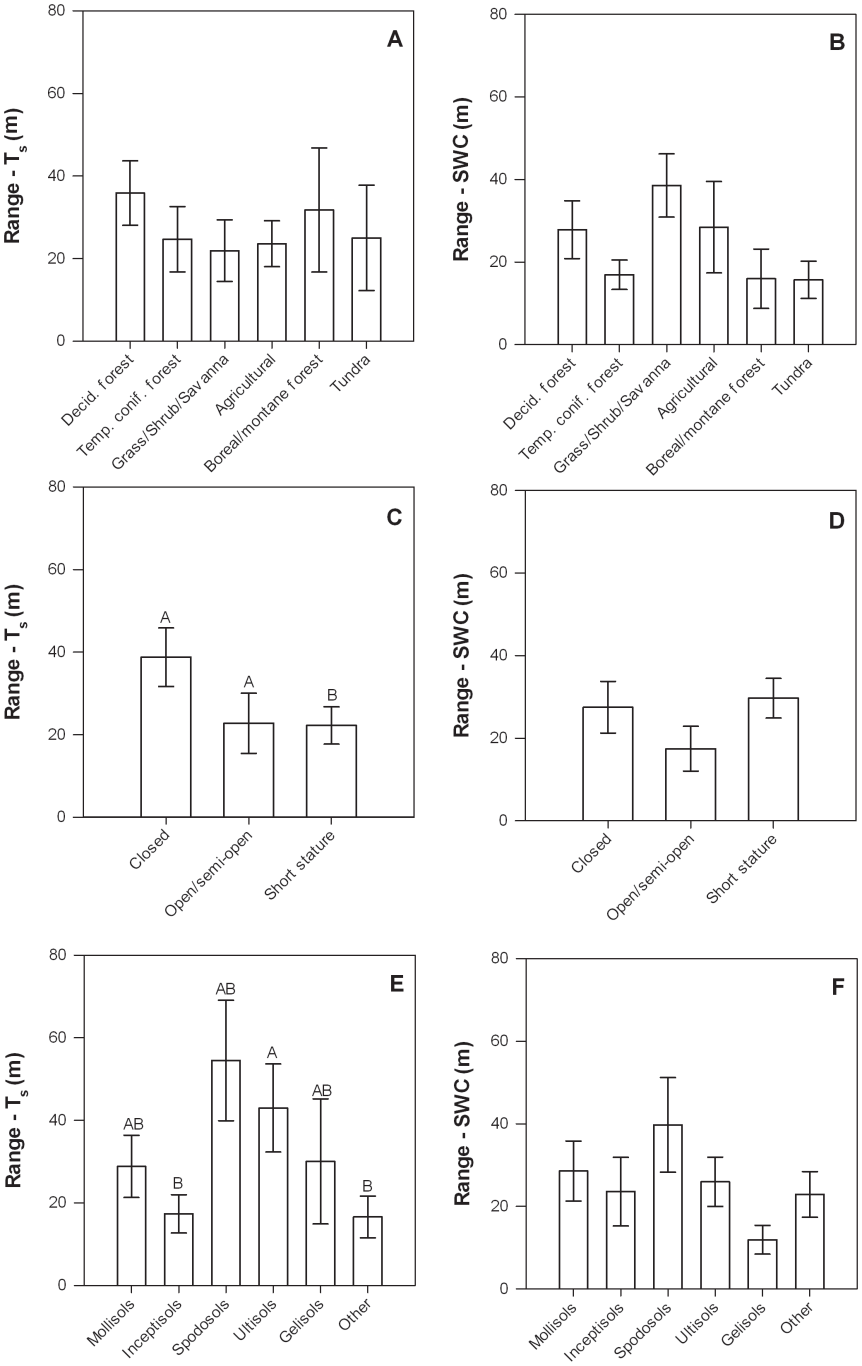
Semivariogram range for T_s_ (left column) and SWC (right column) at each site in relation to ecosystem type (a, b), canopy structure (c, d), and soil type (e, f). Error bars represent ±1 SE. Bars with different letter were significantly different (p<0.05) based on Tukey HSD tests. The letter “A” corresponds to the largest least square mean(s) and subsequent letters (B, C, …) correspond to progressively smaller least square means.

Range values for T_s_ were significantly influenced by canopy structure and soil type, while those for SWC were significantly related to longitude and canopy height (Table 4, Figure 5). T_s_ range values were lower in short stature ecosystems than those in closed canopy or open and semi-open canopy ecosystems, and also lower in Inceptisols and “other” soils than in Ultisols (Figure 5). SWC range values increased with longitude (*i.e.*, increased from west to east) and decreased with canopy height (Table 4).

**Figure 6:**
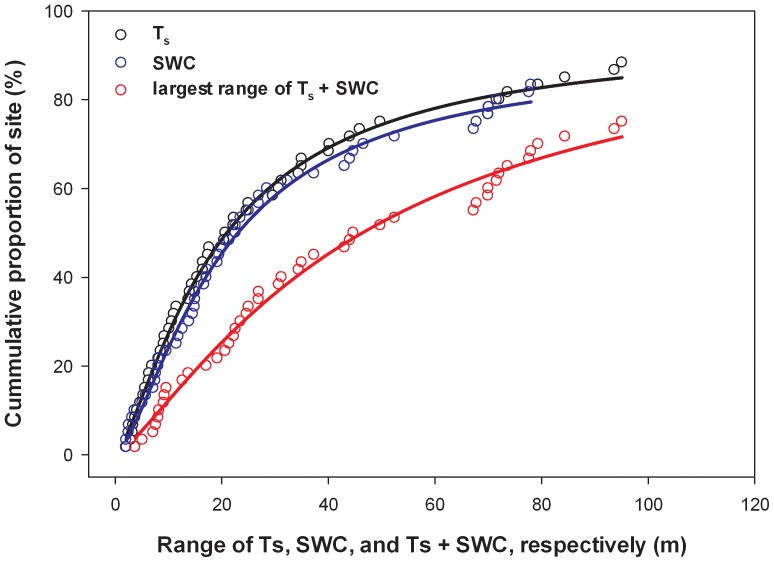
Semivariogram range for T_s_, SWC, and the largest range for T_s_ or SWC at each site versus the cumulative proportion of sites. A logistic curve was fitted to the data with R^2^ of 1.00, 0.99, and 0.99 for T_s_, SWC, and T_s_ and SWC, respectively. Equations for the curves can be found in the results section (Eq. 6a–c).

The relationship between the T_s_ range and the cumulative proportion of sites exhibited a logistic pattern (Figure 6). For example, 88%, 75%, and 55% of sites had a range value for T_s_ of less than 100, 50, and 25 m, respectively. Similar patterns were observed for SWC range values, as well as the largest range value for T_s_ or SWC (*i.e.*, whichever was largest at a site; Figure 6). The equations that described these relationships were,
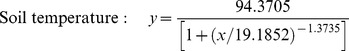
(6a)

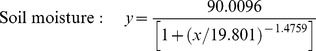
(6b)

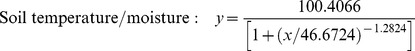
(6c)where, *x* is the range distance expressed in meters, and *y* is the percent of sites that had smaller ranges than *x*.

### Semivariogram sill

**Figure 7:**
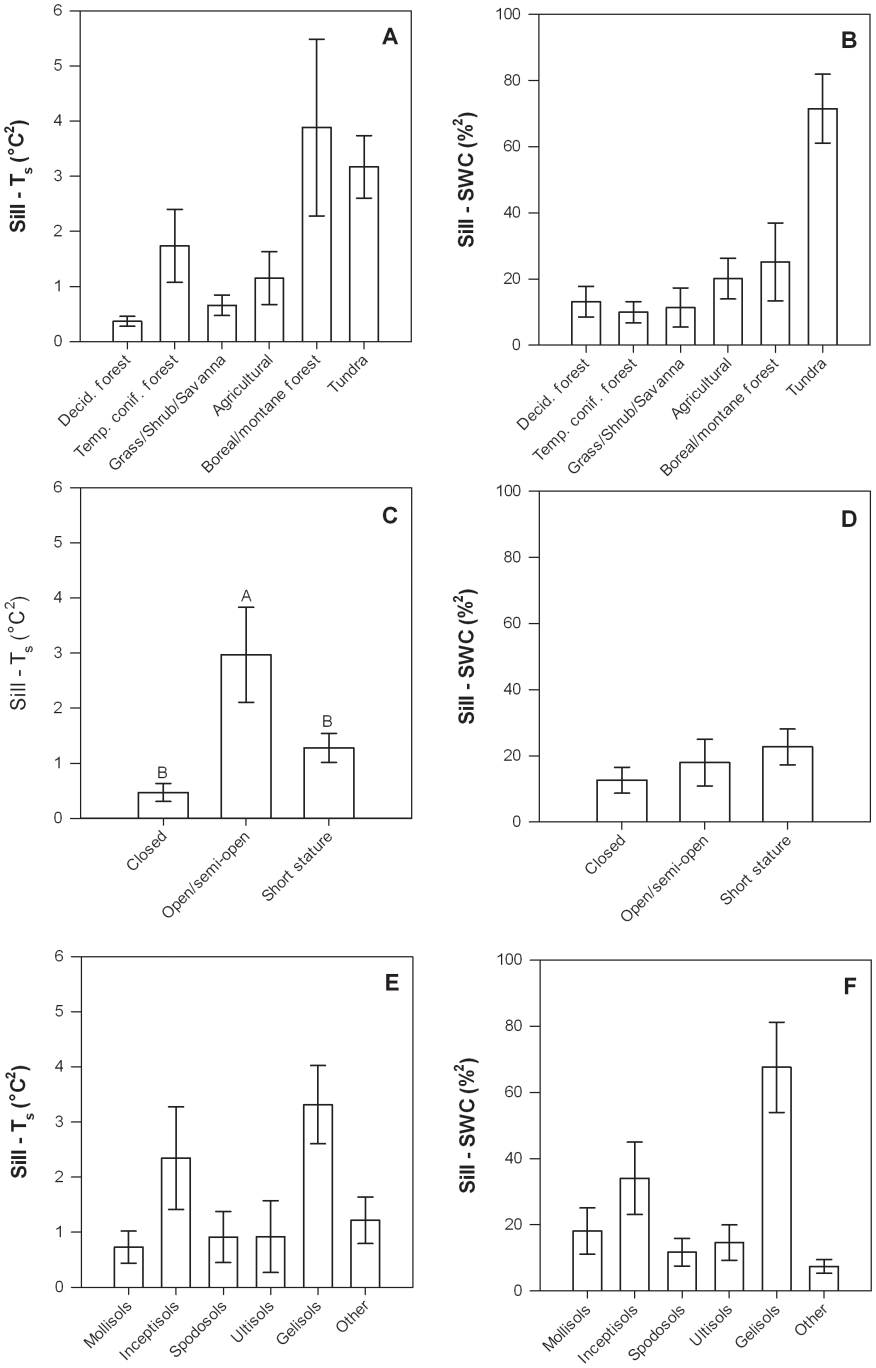
Semivariogram sill for T_s_ (left column) and SWC (right column) at each site in relation to ecosystem type (a, b), canopy structure (c, d), and soil type (e, f). Error bars represent ±1 SE. Bars with different letter were significantly different (p<0.05) based on Tukey HSD tests. The letter “A” corresponds to the largest least square mean(s) and subsequent letters (B, C, …) correspond to progressively smaller least square means.

Sill values for T_s_ and SWC were positively related to one another, suggesting that underlying site properties, in part, mediate the spatial variability in both variables (Table 6, Figure 4b). The sill values for T_s_ were negatively related to longitude (*i.e.*, decreasing from west to east), and mean annual air temperature, positively related to mean T_s_, and higher for open and semi-open ecosystems than for closed canopy or short stature ecosystems (Table 4, Figure 7). The sill values for SWC were also negatively related to longitude and mean annual air temperature, and positively related to mean T_s_ as well as mean SWC (Table 4). Notably, neither ecosystem type nor soil type were significantly related to sill values for T_s_ or SWC (Table 4).

### Semivariogram nugget

The nugget was expressed as a percent of the sill and indicates the proportion of variation that occurred at scales of <1 m. The nugget ranged from 0–81% for T_s_ and 0–96% for SWC, and a paired *t*-test demonstrated that the mean T_s_ nugget (32±3%) was significantly different from the mean SWC nugget (44±3%, p<0.001). Similar to the sill values, T_s_ nugget values were positively correlated with SWC nugget values, indicating that underlying site characteristics mediate sub-meter variability of both parameters (Table 6, Figure 4c).

**Figure 8:**
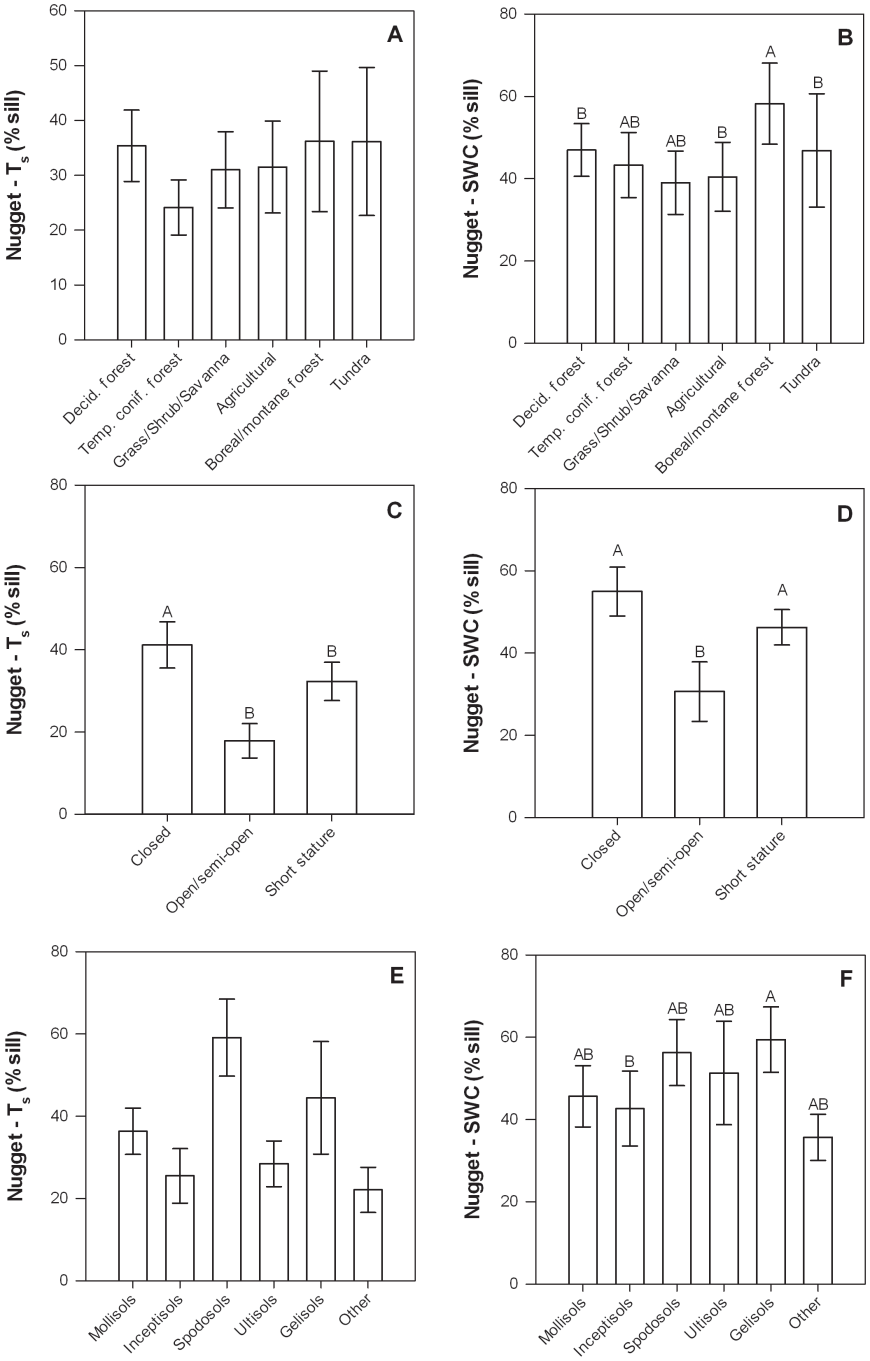
Semivariogram nugget expressed as a percent of the sill for T_s_ (left column) and SWC (right column) at each site in relation to ecosystem type (a, b), canopy structure (c, d), and soil type (e, f). Error bars represent ±1 SE. Bars with different letter were significantly different (p<0.05) based on Tukey HSD tests. The letter “A” corresponds to the largest least square mean(s) and subsequent letters (B, C, …) correspond to progressively smaller least square means.

Nugget values for T_s_ were negatively related to canopy height and mean SWC, and were higher in closed canopy ecosystems than short stature or open/semi-open ecosystems (Table 4, Figure 8). There was also a significant effect of soil type on T_s_ nugget values, however, a subsequent post-hoc test did not reveal significant differences among soil types (Figure 8). Nugget values for SWC were positively related to longitude (*i.e.*, increasing from west to east), negatively related to mean T_s_, as well as being significantly related to ecosystem type, canopy structure, and soil type (Table 4, Figure 8). SWC nugget values were larger in forest ecosystems (particularly boreal/montane forests), and lower in tundra ecosystems, as well as being larger in closed canopy and short stature ecosystems and lower in open/semi-open canopies ecosystems (Figure 8). SWC nugget values were larger for Gelisols than for Inceptisols (Figure 8).

### Relationships among the semivariogram sill, range, and nugget

**Figure 9:**
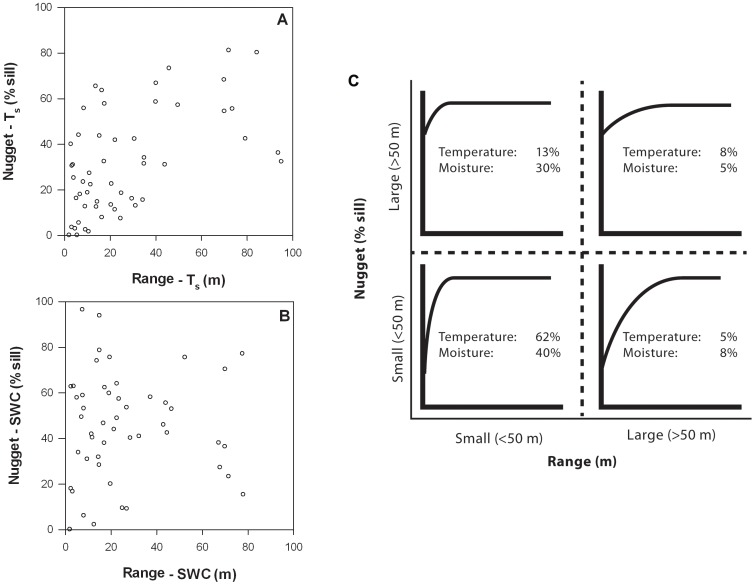
Semivariogram range versus nugget for T_s_ (a) and SWC (b), and the relative occurrence of semivariograms with small (>50 m) or large (>50 m) range values and small (<50% sill) or large (>50% sill) nugget values. The percentages in (c) are based on all 60 sites, including the sites where range values exceeded the maximum distance of the semivariogram model (*i.e.*, 7 sites (12%) for T_s_ and 10 sites (17%) for SWC). As a result, the percentages do not add up to 100%. Since the semivariograms that did not reach an asymptote all had range values that exceeded 50 m, the percentages in the right two quadrants are underestimated by a total of 12 and 17 percentage points for T_s_ and SWC, respectively.

Range values were positively related to nugget values (expressed as a percent of the sill) for the T_s_ semivariograms (Table 7, Figure 9a). However, there was no relationship between range and sill values or nugget and sill values for T_s_ (Table 7). Likewise, range, sill, and nugget values were all unrelated to one another for the SWC semivariograms (Table 7, Figure 9b).

**Table 7:** Spearman correlation coefficients and statistical significance for T_s_ and SWC semivariogram properties.

	Correlation coefficient	P value
T_s_		
Range – nugget	0.53	<0.001
Range – sill	−0.07	0.628
Nugget – sill	−0.15	0.278
SWC		
Range – nugget	0.10	0.492
Range – sill	0.03	0.863
Nugget – sill	−0.17	0.237

The relative abundance of different semivariogram shapes was assessed by assigning them to four categories: small nugget (<50% sill) and small range (<50 m); large nugget (>50% sill) and small range (<50 m); small nugget (<50% sill) and large range (>50 m); and large nugget (>50% sill) and large range (>50 m). For T_s_ the most common combination was a small nugget and small range, which accounted for 37 of the 60 sites (62%; Figure 9c). Small nugget and range values were also the most common combination for SWC, accounting for 24 of the 60 sites (40%). In addition, 18 of the sites (30%) had SWC semivariograms with a large nugget combined with a small range value. For both T_s_ and SWC, it was relatively rare to observe a large range value with either a small or large nugget value (Figure 9c).

### Coefficient of variation and sample size

**Figure 10:**
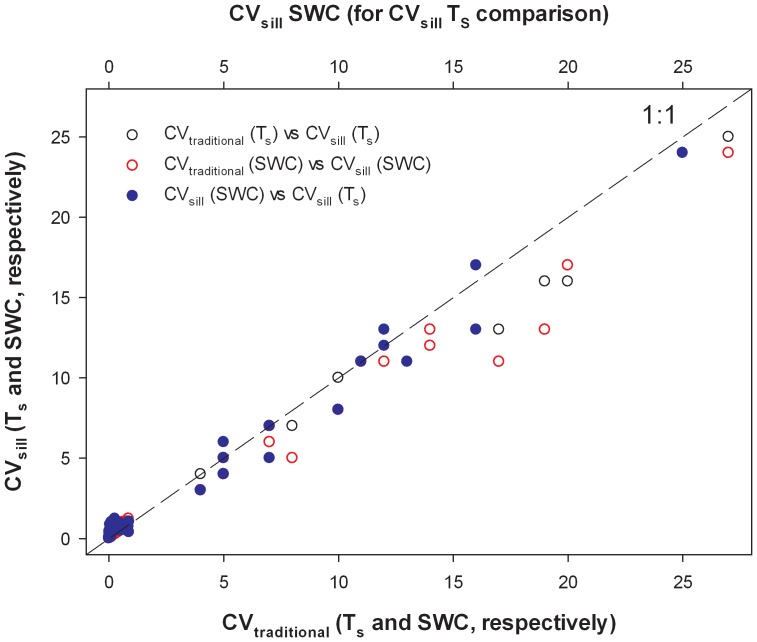
Coefficient of variation calculated by dividing the standard deviation by the mean (CV_Traditional_) versus coefficient of variation calculated using the semivariogram sill (CV_Sill_) for T_s_, SWC, and CV_Sill_ for T_s_ versus SWC. Dotted line is 1∶1.

The two estimates of the coefficient of variation (CV_Traditional_ and CV_Sill_) were strongly and positively correlated with one another (T_s_, r^2^ = 0.96, p<0.001; SWC, r^2^ = 0.95, p<0.001; Figure 10), demonstrating that the semivariograms accurately described the pattern of spatial variability among the sites. However, CV_Tradtional_ values were typically smaller than corresponding CV_Sill_ values for both T_s_ and SWC (p<0.001). This occurred because the traditional method of calculating CV does not account for spatial correlation in data, yet spatial correlation was ubiquitous at our sampling sites (data not shown). Across all sites, CV_Sill_ values were 24±3% and 44±2% larger than CV_Tradtional_ values for T_s_ and SWC, respectively.

The CV_Sill_ values ranged from 0.02 to 0.88, with a mean of 0.16±0.03, and 0.09 to 1.22 with a mean of 0.51±0.04 for T_s_ and SWC, respectively (Table 2). A paired *t*-test demonstrated that T_s_ CV_Sill_ values significantly differed from SWC CV_range_ values (p<0.001), demonstrating that SWC was more variable across space. Similar to the sill values, there was a positive correlation between CV_Sill_ values for T_s_ and SWC (Table 6, Figure 10), again suggesting that variability in these parameters may be controlled by the same site characteristics.

We used a sample size analysis based on CV_Sill_ (Eq. 5) to calculate the number of samples required to estimate T_s_ and SWC to within 10% of the mean with 90% confidence (Table 2). We used CV_Sill_ rather than CV_Traditional_ to calculate sample size because CV_Traditional_ underestimated variability (Figure 10), which in turn would cause the sample size to be underestimated. For example, at Harvard Forest the sample size required to estimate SWC to within 10% of the mean with 90% confidence was 127 when calculated with CV_Traditional_ (0.69), but was 204 when calculated with CV_Sill_ (0.87). Across all the sites, the sample size calculated using CV_Traditional_ underestimated the sample size calculated with CV_Sill_ by a factor of 1.6±0.1 (maximum: 2.6) and 2.1±0.1 (maximum: 3.0) for T_s_ and SWC, respectively. Hereafter, we focus on sample sizes calculated using CV_Sill_.

**Figure 11:**
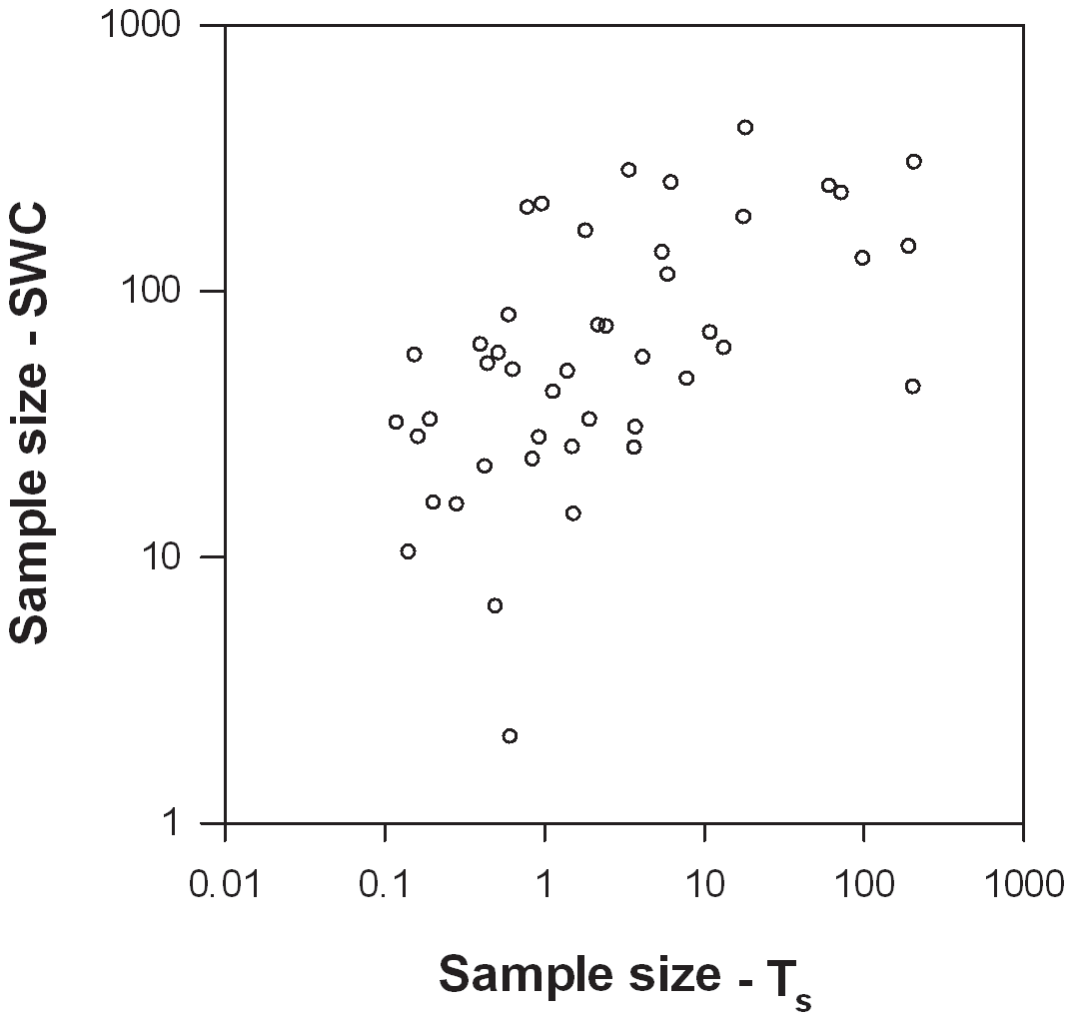
Number of samples required to measure T_s_ to within 10% of the spatial mean with 90% confidence versus the number of samples required to meet the same accuracy requirement for SWC. Values are plotted on a semi-log scale due to large differences in magnitude across the sites.

Since we used the sill to represent the variance in the sample size analysis, the number of samples required assumes that the sample spacing will be larger than or equal to the range value from the semivariogram (*i.e.*, the distance at which the sill is reached). In addition, because the sample size was based on CV_Sill_, both parameters (CV_Sill_ and sample size) exhibited similar patterns. For example, the sample size analyses indicated that the number of samples necessary to meet the accuracy requirement varied from 1 to 211 for T_s_ (mean of 20±7) and from 2 to 405 for SWC (90±13), and a paired *t*-test demonstrated that sample size was significantly larger for T_s_ than SWC (p<0.001), which is similar to findings for CV_Sill_. In addition, the sample size required for T_s_ was positively correlated to the sample size required for SWC (Table 6, Figure 11).

**Figure 12:**
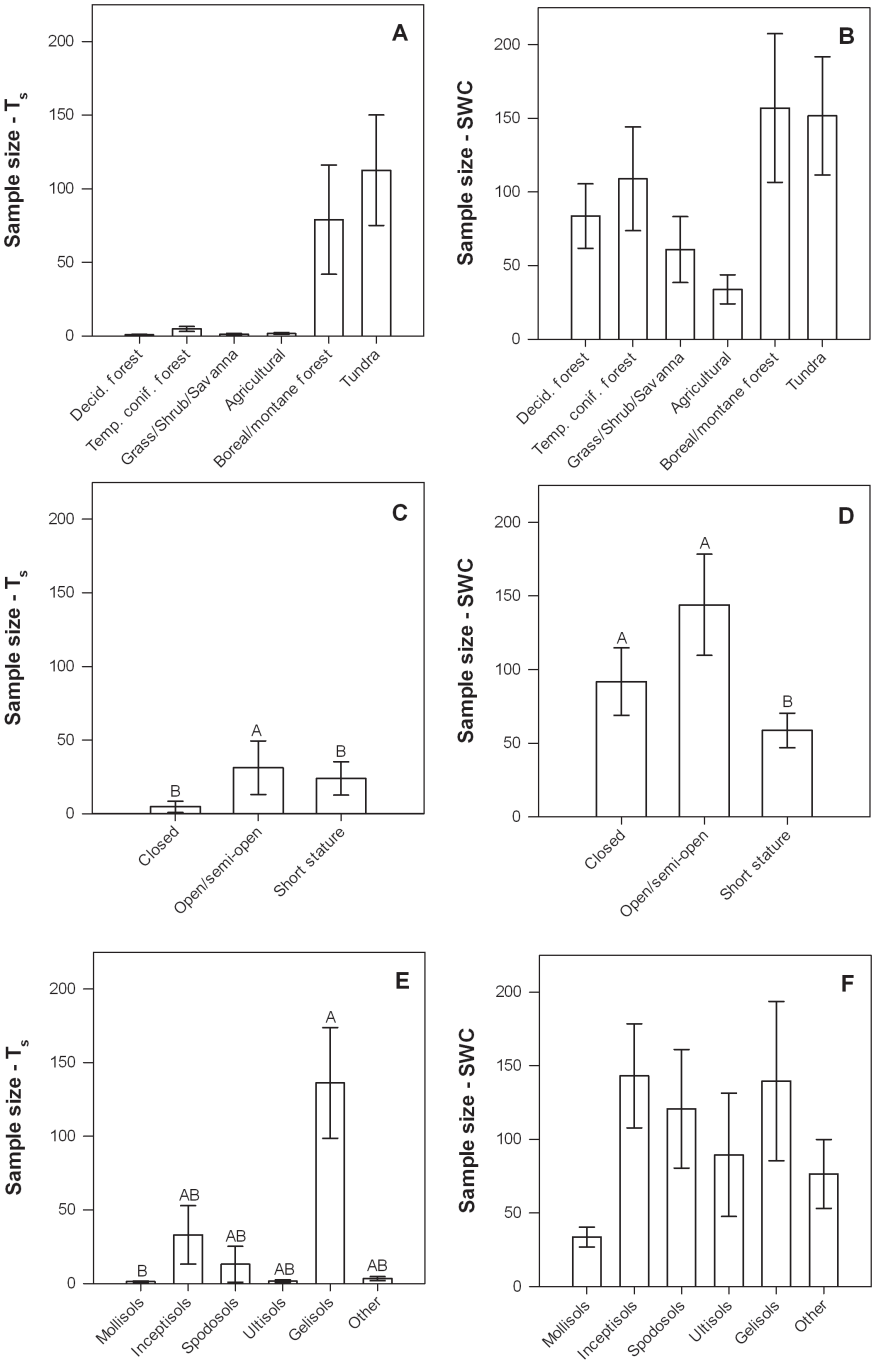
Number of samples to measure T_s_ (left column) and SWC (right column) to within 10% of the spatial mean with 90% confidence at each site in relation to ecosystem type (a, b), canopy structure (c, d), and soil type (e, f). Error bars represent ±1 SE. Bars with different letter were significantly different (p<0.05) based on Tukey HSD tests. The letter “A” corresponds to the largest least square mean(s) and subsequent letters (B, C, …) correspond to progressively smaller least square means.

The number of samples required to estimate T_s_ to within 10% of the mean with 90% confidence was negatively related to longitude (*i.e.*, decreasing from west to east) and mean annual air temperature, and was also influenced by canopy structure and soil types (Table 4, Figure 12). More samples were required in open/semi-open canopy ecosystems than other ecosystems, and in Gelisols than in Mollisols (Figure 12). At 87% of sites, 20 or fewer samples were sufficient to meet the accuracy requirement for T_s_. The remaining 13% of sites that required more samples were all Alaskan sites that shared a number of similarities, including high latitudes, westerly longitudes, low mean annual air temperature (<2°C), low mean T_s_ at the time of sampling (<6°C), low mean annual precipitation (<500 mm), tundra or boreal forest ecosystem types, primarily Gelisols or Inceptisols, low to mid elevations (<1000 m.a.s.l.), and low to mid-vegetation canopy heights (<15 m; Figure 12).

The number of samples required to estimate SWC to within 10% of the mean with 90% confidence was positively related to mean annual precipitation and mean T_s_, negatively related to longitude, mean annual air temperature, mean SWC, and was also influenced by canopy structure (Table 4, Figure 12). More samples were required to meet the accuracy requirement in closed and open/semi-open canopy ecosystems than short stature ecosystems (Figure 12). Unlike T_s_, sites that required a large number of samples (*i.e.*, >100) to accurately measure SWC did not share many similar characteristics, although none had a mean soil moisture of >16% (data not shown).

**Figure 13:**
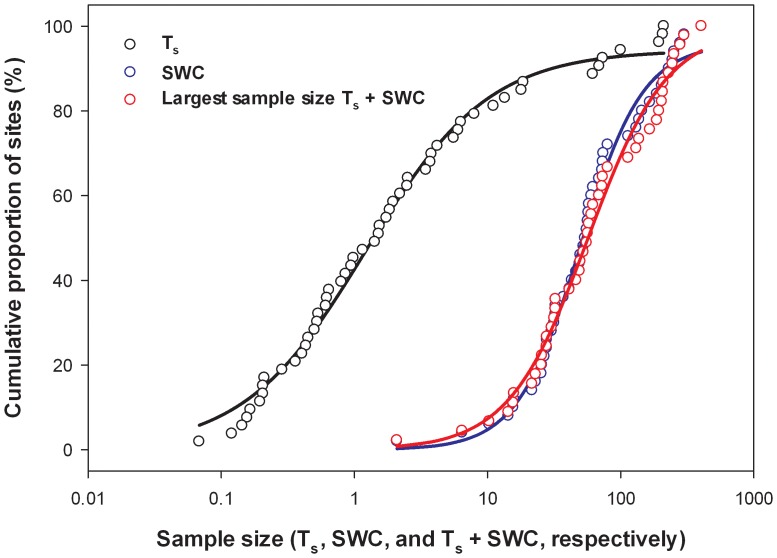
Number of samples required to measure T_s_, SWC, or T_s_ and SWC to within 10% of the spatial mean with 90% confidence versus the cumulative proportion of sites. A logistic curve was fitted to the data with R^2^ = 0.99 for each equation (Eqs. 7a–c). Values are plotted in a semi-log scale due to large differences in magnitude across the sites.

When the required sample size for T_s_ was plotted against the cumulative proportion of sites the relationship could be accurately described by a logistic equation (Figure 13). For example, based on the sample size analyses, one sample was sufficient to estimate T_s_ to within 10% of the spatial mean with 90% confidence at 45% of sites, while 10 samples was sufficient to meet this requirement at 79% of sites. A similar pattern emerged for SWC sample size plotted against the cumulative proportion of sites, although at least an order of magnitude more samples were required than for T_s_ (Figure 13). In addition, the pattern was similar for the number of samples required for T_s_ or SWC (whichever was largest at a site; Figure 13). The equations that described these relationships were,
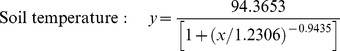
(7a)

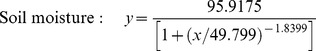
(7a)

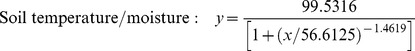
(7c)where, *x* represents a number of samples and *y* is the percent of sites that require *x* (or fewer) samples to estimate the spatial mean to within 10% of the mean with 90% confidence.

### Efficacy of sampling strategies

To estimate the mean T_s_ across the area we sampled at a particular site with the fewest samples, sample spacing should equal or exceed the range value estimated from the T_s_ semiovariogram at that site. This is because samples spaced this distance apart are effectively independent, thereby providing the maximum amount of information from each individual sample. In contrast, if the sample spacing was less than the range value then adjacent samples would be correlated (*i.e.*, each sample would provide less than the maximum amount of information), which meant that more samples would be needed to accurately estimate the spatial mean of T_s_. We emphasize that using the semivariogram range to inform sample spacing is appropriate here because we are only constraining the sampling to accurately estimate the mean. However, this may not be appropriate for studies with different goals.

**Figure 14:**
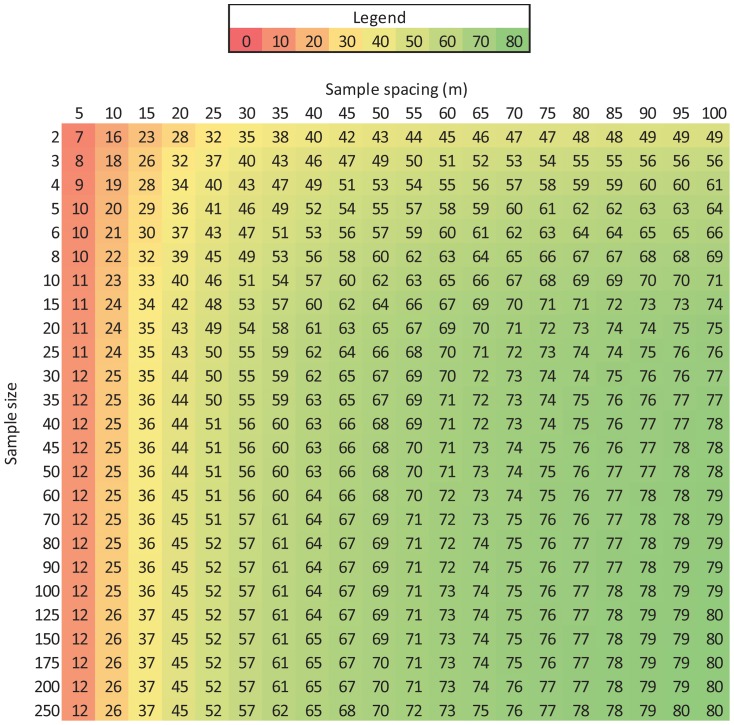
Efficacy of different sampling strategies at measuring T_s_ to within 10% of the spatial mean with 90% confidence at scales of ∼1 ha. Values represent the percent of sites where the corresponding sampling strategy would achieve the accuracy requirement. The x-axis is based on Equation 6a and the y-axis is based on Equation 7a.

**Figure 15:**
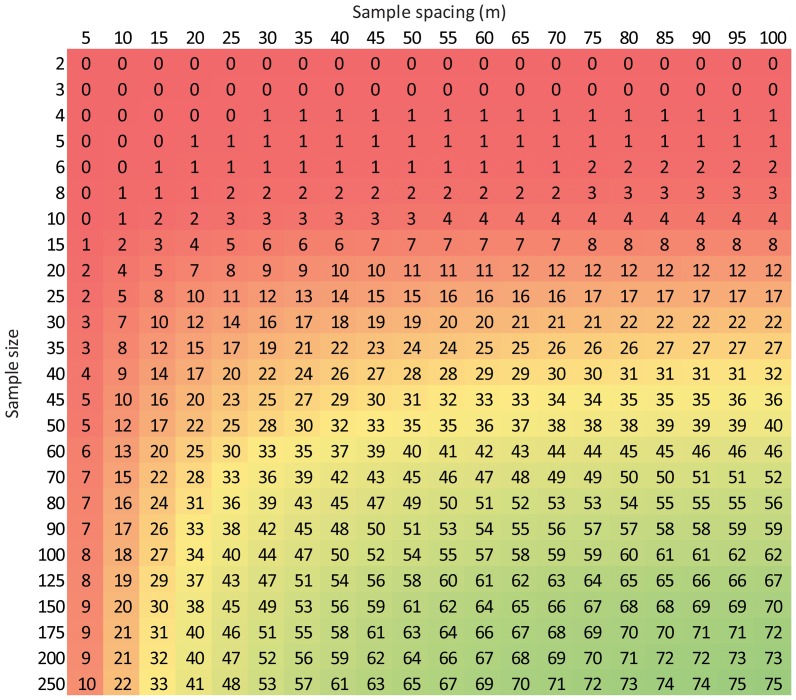
Efficacy of different sampling strategies at measuring SWC to within 10% of the spatial mean with 90% confidence at scales of ∼1 ha. Values represent the percent of sites where the corresponding sampling strategy would achieve the accuracy requirement. The x-axis is based on Equation 6b and the y-axis is based on Equation 7b. Legend in Figure 14.

**Figure 16:**
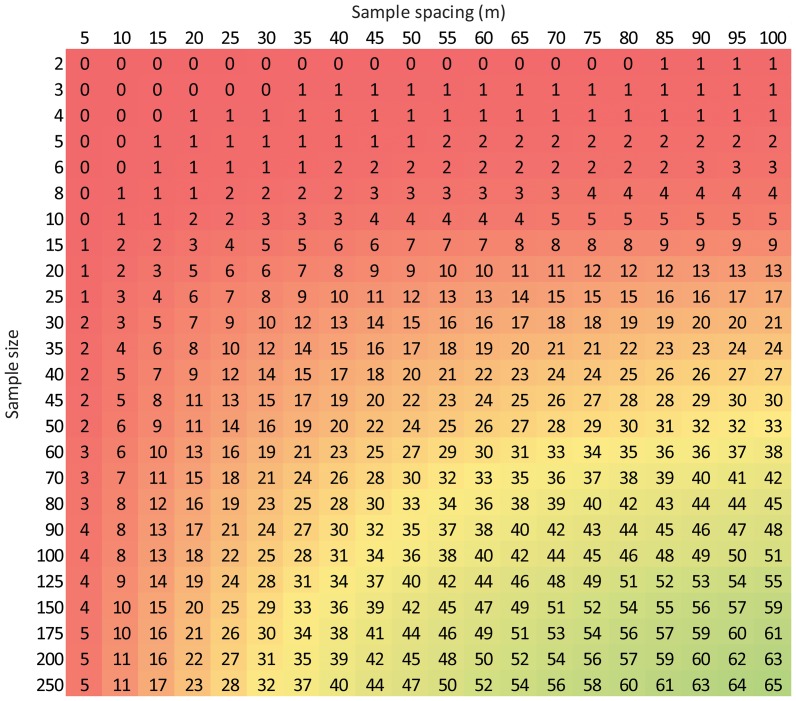
Efficacy of different sampling strategies at measuring T_s_ and SWC to within 10% of the spatial mean with 90% confidence at scales of ∼1 ha. Values represent the percent of sites where the corresponding sampling strategy would achieve the accuracy requirement. The x-axis is based on Equation 6c and the y-axis is based on Equation 7c. Legend in Figure 14.

Since Eq. 6a describes the proportion of sites with a soil temperature range less than a given value and Equation 7a describes the proportion of sites where a given sample size would be required to estimate soil temperature to within 10% of the mean with 90% confidence, we created a matrix that outlines the efficacy of different sampling strategies based on these two equations (Figures 14–16). For example in Figure 14, two temperature measurements spaced 5 m apart would only be sufficient to estimate mean T_s_ to within 10% of the mean with 90% confidence over ∼1 ha (the approximate size of the area we sampled) at 7% of our sites. Interestingly, increasing the number of samples while keeping sample spacing at 5 m barely improved the efficacy of the sampling strategy and this occurs for two reasons, i) because spatial variation in T_s_ is often low (Figure 10), so few samples are needed at most sites (Figure 13), and ii) because only 12% of sites had a range value of 5 m or less (Figure 6). However, if the sample spacing were increased to 100 m, just two samples would be needed to meet the requirement of measuring T_s_ to within 10% of the spatial mean with 90% confidence at half the sites, although common sense dictates that more samples would be preferable (*e.g.*, 5 samples). This matrix can be used as a guide to quantitatively evaluate the efficacy of increasing sample size and/or sample spacing to measure the spatial mean of T_s_ at scales of ∼1 ha at our sampling sites.

Using Equation 6b and 7b, we created a similar matrix to evaluate the efficacy of different sampling strategies for measuring SWC at spatial scales of ∼1 ha (Figure 15). As with T_s_, 2 samples spaced 5 m apart would not be an effective sampling strategy to measure SWC to within 10% of the mean with 90% confidence. Increasing the sample size or sample spacing only slightly improves the efficacy, however, by increasing both the sample size and sample spacing the sampling strategy can become substantially more effective at meeting the accuracy requirement. Figure 15 also demonstrates how a sampling strategy that is effective at a given proportion of sites can be achieved in many different ways. For instance, 150 samples spaced 10 m apart would meet the accuracy requirement for SWC at the same proportion of sites as 60 samples 15 m apart or 30 samples 55 m apart.

Lastly, using Equation 6c and 7c we created a matrix to evaluate the efficacy of measuring both T_s_ and SWC to within 10% of the mean with 90% confidence at spatial scales of ∼1 ha (Figure 16). I n this case, more samples spaced further apart were always required to meet the accuracy requirement at a given proportion of sites than for T_s_ or SWC alone.

## Discussion

We characterized the spatial structure of variability in soil properties over ∼1 ha at 60 sites throughout the US. Our study included nearly an order of magnitude more sites than any previous study that examined the spatial variation in soil properties, and as a result, allowed us to derive some empirical rules to describe the observed variability in soils. Moreover, we were able to evaluate the efficacy of different sampling strategies (*i.e.*, sample size and sample spacing) at measuring the spatial mean of T_s_ and SWC, which can guide future soil studies. We emphasize that our guidelines for sampling strategies are designed to accurately estimate the spatial mean of a soil property, and as a result, our guidelines may not be suitable for studies with different goals. Because our sites were broadly representative of US geography and ecology (*i.e.*, including every major terrestrial ecosystem type and soil type), our findings are broadly applicable to US soils, and to a lesser extent other regions of the world.

### Variability

Generally, variability was larger for SWC than T_s_, which resulted in a larger sample size required to measure SWC to within 10% of the spatial mean with 90% confidence than for T_s_. This is consistent with other studies that have also observed larger CV values for SWC than T_s_ [23], [24], [58]–[60]. We did not specifically set out to determine the sources of variability, or why variability was larger for SWC than T_s_, but a likely explanation is that water is more mobile than heat, and as a result more sensitive to microtopography (*i.e.*, water drains from hummocks to depressions, whereas heat does not), and is actively transported by plants (*i.e.*, transpiration induced uptake and hydrologic lift), resulting in larger variability at this spatial scale.

Despite generally larger spatial variability in SWC than T_s_, (*i.e.*, sill and CV_Sill_), T_s_ and SWC were positively correlated suggesting that they were likely controlled by similar site properties at the scale we sampled. Indeed, ANOVAs also indicated that sill values for both measures decreased with increasing longitude (*i.e.*, from west to east) and mean annual air temperature, while they increased with mean T_s_. In addition, among the sites sill values for temperature and moisture increased with mean T_s_ and SWC, respectively, exhibiting the commonly observed pattern of increased variability with increasing mean values, *i.e.*, heteroscedastic [61]. Similarly, data presented by Brocca et al. [12] shows that the standard deviation for SWC increased from the driest site (MON, mean ± SD: 22.8±2.0) to the wettest site (LEC, 38.5±3.0) for seven Italian sites. In contrast, Western et al. [26] found no clear relationship between SWC and variance at locations in Australia and New Zealand, while data reported by Famiglietti et al. [25] show a negative relationship among six sites in Oklahoma. These studies however, differ in their design (*e.g.*, spatial scale, sampling frequency, geographic region), making it difficult to determine why different patterns were observed among the studies. But one explanation could be that the driest sites in the other studies had a mean SWC of between 10% and 23%, whereas half of our sites had a mean SWC of <10% (all of which had relatively low semivariogram sill values) and previous studies that have monitored SWC over time at individual sites often observed that low mean SWC coincided with relatively low variability, *e.g.*, [13], [14]. We studied the relationship between the mean and variability (*i.e.*, sill) across space, whereas most previous studies have investigated this relationship at individual sites across time and have often (but not always) observed the variance or standard deviation increasing with intermediate SWC and decreasing at low and high SWC [12]–[14], [20], [26].

Ecosystem type did not significantly influence sill values for either T_s_ or SWC, despite the common assumption that agricultural soils were less variable than soils in other ecosystems [4], [11]. Similarly, Robertson et al. [11] were surprised by the large variability in a wide range of soil properties from a soybean field that visibly appeared homogeneous, supporting the notion that agricultural ecosystems are typically more variable than many researchers appreciate.

The ANOVA models left 43% and 30% of the variability in T_s_ and SWC sill values unexplained for, respectively, indicating that other site properties (*i.e.*, besides the ones we measured) also are sources of variability. In part this may relate to vegetation canopy structure at the sites. For example, soil beneath canopy gaps would receive both more radiation and precipitation inputs than soil beneath a closed-canopy, which would increase spatial variability in both measures at a site [62]. Indeed, T_s_ sill values were significantly larger in open/semi-open ecosystems (*i.e.*, sites with high variability in vegetation cover) than in closed-canopy or short stature ecosystems, but this pattern did not occur for SWC. In a rangeland ecosystem, diurnal maximum T_s_ at 5 cm were found to be much lower beneath sagebrush plants (10°C) than in interspaces between plants (17°C), indicating that canopy structure strongly influenced spatial variability in T_s_ [63]. Similar results have been found with SWC [64], [65]. In addition, previous studies have shown that topography influences the spatial variability of SWC [12], [66], [67], which may also account for some of the unexplained variation found here.

### Sample size

We estimated the number of samples required to measure T_s_ and/or SWC to within 10% of the spatial mean with 90% confidence at each site for prospective purposes (*i.e.*, to inform future study designs). This represents a conservative number of samples for most studies, as this level of accuracy is often deemed more than sufficient and some have supported even lower accuracy requirements [4], [5]. Of course each study should evaluate their required accuracy, and if these criteria were relaxed, design elements like sample size could be reduced.

Previous estimates of the sample size required to measure soil properties to a given accuracy have often assumed that their data was not spatially correlated [12], [13], [14], [24]. However, our data, and data from many other studies [9], [18], [23], [24], [26], [59], [68]–[72], show that spatial correlation is ubiquitous for T_s_ and SWC. Since CV_Traditional_ did not account for spatial correlation (*i.e.*, it assumes that all measurements are independent regardless of how closely they are spaced) it underestimated the true variability in the data, causing the required sample size to be underestimated [73]. In contrast, CV_Sill_ did account for spatial correlation, therefore, it reliably estimated the variability in the data and is appropriate for calculating the required sample size. Had we used CV_Traditional_ rather than CV_Sill_ to calculate sample size, we would have substantially underestimated the required sample size (*i.e.*, by 60% for T_s_ and 109% for SWC when averaged across all sites). As a result, caution should be used when relying on sample size estimates that assume no spatial correlation in T_s_ and SWC data.

Other methods can be used to estimate the number of samples required to accurately estimate the spatial mean of a soil property. For example, previous research has shown that fewer samples are sufficient to meet an accuracy requirement if the spatial mean is calculated by kriging, rather than by averaging the data [74]–[77]. However, the kriging method requires knowledge of the semivariogram at the sampling site, which is frequently unknown. Moreover, our data shows that it is not possible to reliably estimate the key features of T_s_ and SWC semivariograms (*i.e.*, sill, nugget, and range) based on the site characteristics we studied. Similarly, the temporal stability (or rank stability) approach and the random combination method can also be used to reduce the required sample size, but these approaches require detailed knowledge of spatial variation of soil properties at the sampling site, which is also often unknown [14], [24], [77], [78]. It should be noted that these approaches address a slightly different goal (*i.e.*, estimating the number of samples required to estimate mean soil moisture over a number of time steps) than the statistical method to calculate sample size, which focuses on a single time step. Because of these limitations, we calculated the required sample size using the classical statistical approach (Eq. 5) to allow our findings to be more easily applied to sampling designs at sites where the spatial structure of variation may be unknown.

Spatial variability in T_s_ was so low at many sites that the sample size analyses indicated that even just one sample was sufficient to meet our accuracy requirement (although common sense dictates that more samples would be preferable), while 10 samples were sufficient at 79% of sites, and 100 samples were sufficient at all but 3 sites. In contrast, SWC often required at least an order of magnitude more samples to meet the accuracy requirement at a similar proportion of sites (*e.g.*, 100 samples were sufficient at 72% of sites). Because more samples were required to meet our accuracy requirement for SWC than T_s_ at almost every site, the sample size needed when examining both T_s_ and SWC was very similar to that found for SWC alone.

The large sample sizes needed to accurately measure the spatial mean SWC based on our findings differ substantially from previous studies that assumed SWC was not spatially correlated [12]–[14]. For example, Brocca et al. [12] reported that 15 or fewer samples would be needed at 4 out of 8 sites to measure SWC to within ±2% volumetric with 95% confidence, and at most 40 samples would be needed to meet this accuracy requirement at any of the sites. We largely attribute this difference to accounting for spatial correlation in our estimates of sample size, but other factors may also play a role including that many previous studies have focused on agricultural and grassland sites, which tend to require smaller sample sizes (Figure 13). In addition, at site with a mean SWC of <20%, our relative error threshold (10% of the mean) is more stringent than the 2% absolute error that has often been used in previous SWC studies. We are not aware of previous studies that have estimated the sample size required to measure T_s_ to a given accuracy, but our data suggests these estimates will only be reliable if they account for spatial correlation.

Based on our finding, 20 samples would be more than sufficient to meet the accuracy requirement for T_s_ at most sites, although Alaskan sites typically required many more samples to meet the same requirement. As a result, measuring T_s_ across space in a temperate or sub-tropical ecosystem one might reasonably assume that 20 samples would be sufficient (assuming appropriate sample spacing), while working at high latitude (>50°N) one would likely need an order of magnitude more samples to have confidence in meeting the same accuracy requirement. Sites where a large number of samples were required were more closely associated with high latitudes, westerly longitudes, and cold mean T_s_ at the time of sampling (<6°C).

There are plausible reasons why sites with both low mean T_s_ and high latitude would result in the need for large sample sizes. First, sites with low mean T_s_ statistically result in high CV values because the equation divides by the mean T_s_ (see Eq. 4), which in turn results in large sample sizes (see Eq. 5). Second, the low angle of the sun at high latitude sites, which results in large differences in shading (*i.e.*, radiation inputs) of locations north or south of an obstacle (*e.g.*, tree trunk, tussock, or rock) will spend a large amount of time shaded by the obstacle and when unshaded, the sun will be at a low angle (*i.e.*, low radiation inputs), and collectively cause large spatial variability in T_s_. The converse is true at lower latitude sites where shading by an obstacle will be much less than at high latitude sites creating less spatial variation in T_s_ and in turn results in fewer samples necessary to meet the accuracy requirement. Indeed, the positive relationship that we observed between T_s_ sill values and latitude lends additional support to the first hypothesis, suggesting that high latitudes, as well as low mean T_s_ values, both result in large sample sizes. In contrast, we are not aware of a plausible reason why westerly longitudes should be expected to increase the number of samples required, instead it seems likely that relationship between longitude and the number of samples is an artifact of US geography, since all high latitude sites in the US are located in Alaska, which is located further west than most of the temperate and sub-tropical US. Thus, it appears that the low mean T_s_ and high latitudes, not westerly longitudes, of the Alaskan sites resulted in the large sample sizes for T_s_ required at those sites.

Unlike T_s_, the sample sizes required to accurately measure spatial SWC were only weakly related to site characteristics making it difficult to estimate a sample size based solely these variables. However, it is notable that a sample size of 100 was sufficient at most sites (72%), while sites that needed >100 samples all had mean SWC of <16%, and sites that needed >250 samples had mean SWC of ≤11%. Similar to T_s_, the reduction in the required sample size at high mean SWC is presumably partly due to the fact that low mean SWC results in large CVs because this is calculated by dividing by the mean SWC (see Eq. 4), which in turn causes large sample sizes to be needed to accurately estimate the spatial mean (see Eq. 5).

The large sample size that was required to accurately estimate the spatial mean SWC across ∼1 ha at most sites is beyond the scope of many research projects. However, the recent development of sensors that aim to measure SWC at scales of hundreds and thousands of m^2^ may hold promise for estimating mean SWC at this scale with just 1 or a handful of sensors [79], [80] and may represent a more cost-effective way of meeting the accuracy requirement than deploying tens or hundreds of sensors that each measure SWC in a small volume of soil.

### Sample spacing

The number of samples required to meet our accuracy requirement assumes that the samples are spaced at least at the distance of the range in order to avoid correlation and pseudoreplication [1]. The mean range for both T_s_ and SWC was ∼27 m. Similar range values have been observed for T_s_ and SWC in other studies that sampled at a similar scale [8], [18], [20], [59], [71], [81], demonstrating that range values of tens of meters are the norm for temperature and moisture at this scale.

Unlike the sill values, the range values for T_s_ and SWC were not correlated, indicating that they are controlled by different site properties. The ANOVAs supported this conclusion, showing that range values for T_s_ were related to canopy structure and soil type, whereas for SWC they were related to longitude and canopy height. However, the ANOVA models left 60% and 82% of the variability in range values unexplained for T_s_ and SWC, respectively, indicating that we did not measure the most important factor(s) that control the spatial structure of variability. As a result it is difficult to determine what controls the spatial structure of variability at our sites, but potential factors may include microtopography, canopy gap spacing, and variation in soil texture.

Since we cannot reliably predict the semivariogram range for T_s_ or SWC based on the site characteristics that we measured, a researcher wishing to measure T_s_ and/or SWC at scales of ∼1 ha could use Equations 6a–c to estimate if a given sampling spacing would likely be sufficient (*i.e.*, larger than the range value). For instance, a sample spacing of 5 m would likely be deemed insufficient since only ∼12% of sites had a smaller T_s_ or SWC range, whereas a sample spacing of 40 m may be considered more appropriate since ∼2/3 of sites had a smaller T_s_ or SWC range than this. This sample spacing could then be coupled with the estimated number of samples required based on latitude and mean T_s_ (for spatial measurements of temperature) and/or mean SWC (for spatial measurements of moisture) to develop a site-specific sampling strategy.

### Sampling strategies

Figures 14–16 can guide a sampling design. These figures clearly demonstrate the potential benefit of increasing sample number and/or sample spacing at the spatial scale we sampled. In particular, there are many different ways of achieving a sampling strategy that is likely to meet our accuracy requirement at a given proportion of sites (Figures 14, 15, 16). This demonstrates a particularly pertinent finding that increasing sample spacing is as important as increasing sample number to achieve a given sampling efficacy. This is significant because doubling the samples size often creates much more work and/or expense than simply doubling sample spacing.

Several existing networks that monitor T_s_ and SWC (*e.g.*, USDA Soil Climate Analysis Network (SCAN) and the US NOAA Climate Reference Network (USCRN)) use sampling designs that would likely require supplemental sampling effort in order to accurately estimate the spatial (∼1 ha) mean of these properties at most sites (rf. Figures 14, 15, 16). To illustrate this point, USCRN deploys 3 T_s_ and SWC sensors 5.2 m apart in a range of depths at >100 sites throughout the US (M Palecki, pers. comm.), while SCAN deploys 1 T_s_ and SWC sensor at several depths at sites throughout the US [33]. Both the number of sensors and their spatial distribution would be inadequate to estimate these quantities within 10% of the mean with 90% confidence. To highlight the utility of our data, an effective way to increase the local scale (∼1 ha) spatial accuracy in this case would be to increase sample spacing and add more SWC sensors (rather than T_s_ sensors).

**Figure 17:**
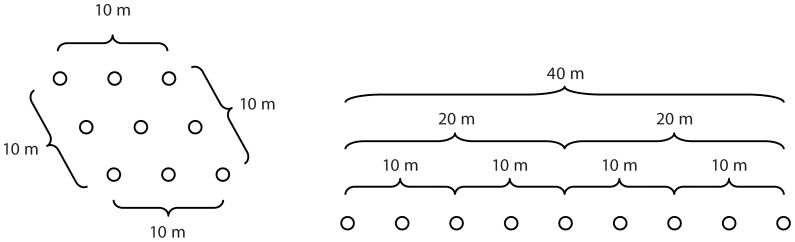
Nine sampling points (open circles) spaced 5 m apart and arranged (left panel) in a 3×3 grid and (right panel) along a transect. Besides 9 samples spaced 5×3 grid also includes 4 samples spaced 10 m apart, while the transect also includes 5 samples spaced 10 m apart, 3 samples spaced 20 m apart, and 2 samples spaced 40 m apart.

Using the results found here, we have constructed a simplified matrix to inform researchers on the efficacy of different sampling designs (Figures 14, 15, 16), given the caveat that they do not account for information provided by samples spaced at distances less than the range; 2) recognize that one T_s_ sample (*i.e.*, 0 m sample spacing) is sufficient at several sites; and 3) recognize that in practice a sampling design operates at multiple spatial scales, *e.g.*, 9 samples spaced 5 m apart and arranged in a 3×3 grid also includes 4 samples spaced 10 m apart (Figure 14). Similarly, 9 samples spaced 5 m apart along a transect also includes 5 samples spaced 10 m apart, 3 samples spaced 20 m apart, and 2 samples spaced 40 m apart (Figure 17). Nonetheless, these figures provide an empirically-derived quantitative starting point for developing robust soil sampling strategies.

In addition to the limitations associated with Figures 14, 15, 16, researchers interested in using our findings to guide their sampling strategy should also bear in mind the conditions under which our data were generated. For example: 1) the sites were typically dominated by a single vegetation type, therefore our findings may not adequately capture variability at locations that cross major vegetation ecotones; 2) data were collected during growing season, so may not represent other seasons; 3) since our sampling typically occurred on a single day, which may have represented anomalous conditions (*e.g.*, usually hot, cold, wet, or dry), we expect that the general patterns we observed across multiple sites are more reliable than data from an individual site; 4) our data are representative of the scale that we sampled (∼1 ha) and may not be applicable at substantially smaller or larger scales; and 5) our data are based on surface soil measurements, which typically exhibits larger spatial variability than deeper soils [63], [82].

### Emergent continental scale properties

Soil forming processes, parental material, time, climate, weathering type and rates, and biological activity have been recognized in soil classification schemes [83], [84]. But because soil genesis is difficult to measure directly, the current US soil taxonomy, rf. [84] relies on soil properties (pedology) that only implies genesis. This classification system provides an underlying framework of physical mechanisms that contribute towards the spatial correlation that we attempt to quantify. Here, we have shown new evidence of emergent continental scale soil properties, *i.e.*, how ecosystem scale spatial variability is patterned across the US. Granted, this variability can be either interpreted directly as functions of soil temperature and moisture, or by proxy for other soil properties. We also fully recognize that there are a variety of local scale controls on this variability, *i.e.*, canopy structure, canopy height, etc. (that mostly control the temperature and/or water microclimate), but this does not preclude the existence of continental scale patterns that we identified, as these influences manifest themselves at smaller spatial resolutions.

Continental scale patterns included: 1) SWC range values increased with longitude (*i.e.*, increased from west to east), 2) sill values for T_s_ and SWC were negatively related to longitude (*i.e.*, decreasing from west to east), 3) nugget values for SWC were positively related to longitude (*i.e.*, increasing from west to east), and 4) range values for T_s_ increased from inceptisols (younger soils) to spodosols (intermediate age) to ultisols (older soils), suggesting that the spatial structure of variability is in part related to soil development. Such patterns could not have been identified from previous studies since they involved too few sites (≤6), and a meta-analysis approach was not possible due to large methodological inconsistencies among the studies (*i.e.*, different sampling scales, measurement methods, sampling depths, etc).

## Conclusions

We characterized the spatial variability of soil properties at 60 US sites to inform NEON's sensor-based soil sampling strategy. In addition, we developed quantitative guidelines that can be used to inform soil sampling decisions throughout the US and elsewhere. While our data can be used as a guide, the most reliable way to generate a robust sampling design would be to conduct a site specific assessment of variability in soil properties on several occasions during the relevant season and at the relevant scale, as others have previously suggested [4], [5]. Indeed, as Klironomos *et al.* [5] highlighted, although a preliminary study of spatial variation may seem like a large amount of extra work, it significantly contributes towards avoiding a sampling strategy that does not provide data that are both necessary and sufficient to meet the design constraints. However, since soil researchers have only rarely followed these recommendations, we also present our findings as a useful tool to help guide future soil sampling designs.

## Supporting Information

Figure S1 **Data collected across (a) space can be used to construct a (b) semivariogram, which describes the relationship between semivariance (** ***i.e.*** **, half the variance) and distance.** A typical sampling layout used in this study is shown in (a). In addition to collecting data at each point shown on the graph, data were also collected at −0.3 m and −0.3 m from each point along the axis of each transect. The three components that describe the shape of the (b) semivariogram are the sill, nugget, and range. The sill represents the maximum semivariance that is encountered at a site and is equivalent to half the variance in the data set used to create the semivariogram. The nugget represents the variance that exists at spatial scales smaller than the minimum sampling distance, as well as sampling error. The range represents the distance beyond which samples are effectively independent at the scale sampled.
(TIF)
