# Supplementary figures and images for: Spatial Variation in Soil Properties among North American Ecosystems and Guidelines for Sampling Designs
Source: PLoS One. 2014 Jan 17;9(1):e83216. doi: 10.1371/journal.pone.0083216 (PMC3894938; doi:10.1371/journal.pone.0083216)

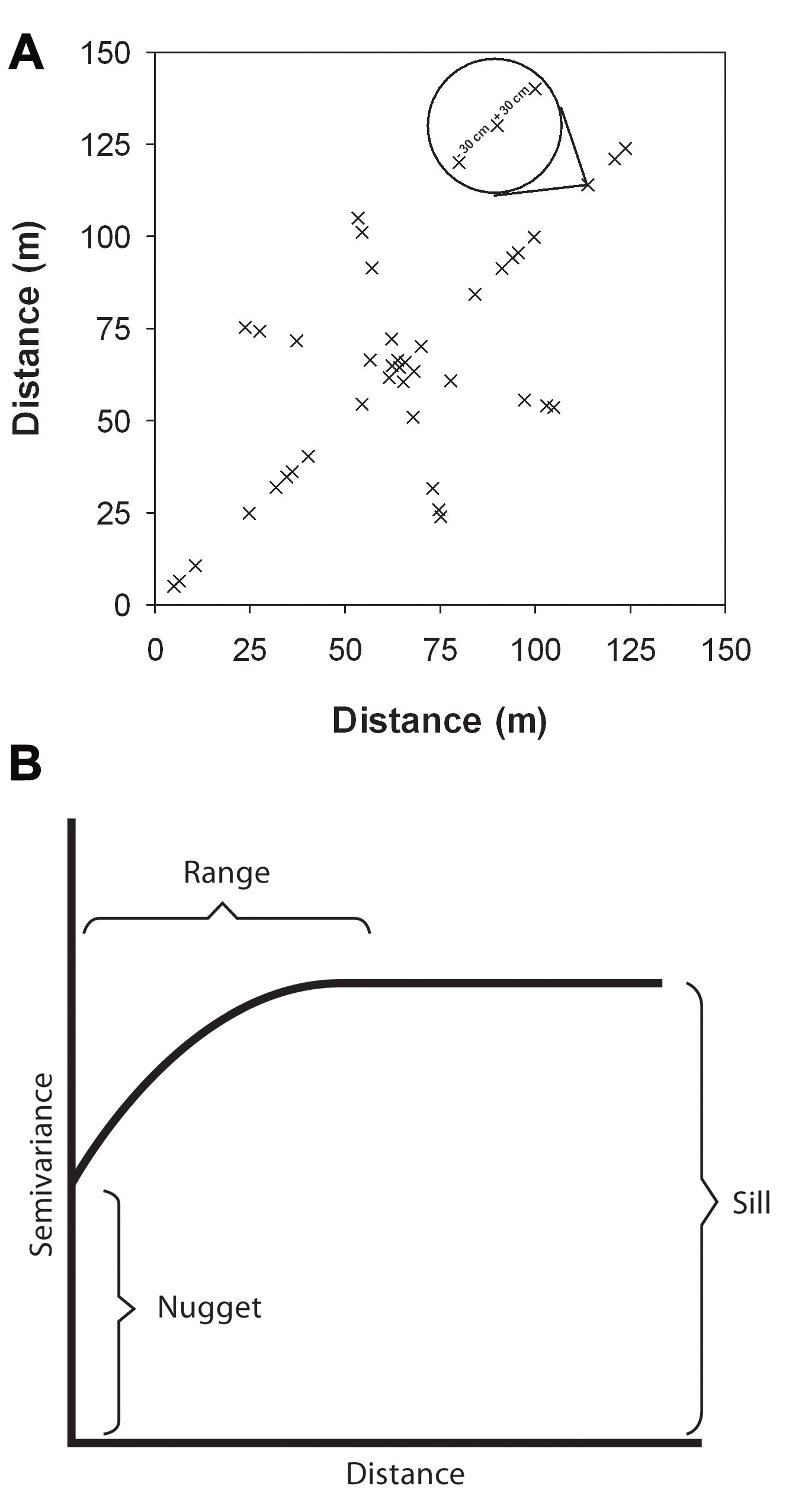

Supplement: Figure S1 — Data collected across (a) space can be used to construct a (b) semivariogram, which describes the relationship between semivariance ( i.e. , half the variance) and distance. A typical sampling layout used in this study is shown in (a). In addition to collecting data at each point shown on the graph, data were also collected at −0.3 m and −0.3 m from each point along the axis of each transect. The three components that describe the shape of the (b) semivariogram are the sill, nugget, and range. The sill represents the maximum semivariance that is encountered at a site and is equivalent to half the variance in the data set used to create the semivariogram. The nugget represents the variance that exists at spatial scales smaller than the minimum sampling distance, as well as sampling error. The range represents the distance beyond which samples are effectively independent at the scale sampled. (TIF) [file pone.0083216.s001.tif]
